# e-Learning, Distance Education, and Virtual and Augmented Reality in Orthopedic Training: European Cross-Sectional Survey of Trainee Acceptance Guided by the Technology Acceptance Model and Unified Theory of Acceptance and Use of Technology

**DOI:** 10.2196/79418

**Published:** 2026-07-10

**Authors:** Adam Tibor Schlegl, Marko Ostojić, Ines Unterfrauner, Gianluca Ciolli, Panayiotis D Megaloikonomos, Vasilios G Igoumenou, Michele Mercurio, Thomas Stark, David Kordic, Martijn Dietvorst, Filipe Lima Santos, Viktória Nyakas, Luca Tóth, András Komócsi, Péter Maróti, András Matuz

**Affiliations:** 1 Department of Orthopaedics Medical School University of Pecs Pecs Hungary; 2 Medical Skills Education and Innovation Centre Medical School University of Pecs Pecs Hungary; 3 Traumatology Department University Hospital Sisters of Mercy Zagreb Croatia; 4 Osteon Orthopedics and Sports Medicine Clinic Mostar Bosnia and Herzegovina; 5 Department of Orthopedics Balgrist University Hospital University of Zurich Zurich Switzerland; 6 Department of Orthopaedics and Traumatology Agostino Gemelli University Polyclinic Rome Italy; 7 Università Cattolica del Sacro Cuore Rome Italy; 8 First Department of Orthopaedic Surgery Attikon University General Hospital National and Kapodistrian University of Athens Athens Greece; 9 Department of Spine Surgery Medius Klinik Nürtingen Nürtingen Germany; 10 Department of Orthopedic and Trauma Surgery "Renato Dulbecco" University Hospital Magna Graecia University Catanzaro Italy; 11 Orthopedics and Trauma Surgery Clinic University Hospital of Basel Basel Switzerland; 12 Department of Orthopaedics University Clinical Hospital Mostar Mostar Bosnia and Herzegovina; 13 Department of Orthopaedic Surgery and Trauma Máxima Medisch Centrum Eindhoven The Netherlands; 14 Serviço de Ortopedia e Traumatologia Centro Hospitalar de Vila Nova de Gaia Vila Nova de Gaia Portugal; 15 Department of Neurosurgery Medical School University of Pecs Pecs Hungary; 16 Institute for Translational Medicine Medical School University of Pecs Pecs Hungary; 17 Heart Centre Medical School University of Pecs Pecs Hungary; 18 3D Printing and Visualization Centre Medical School University of Pecs Pecs Hungary; 19 Department of Behavioural Sciences Medical School University of Pecs Pecs Hungary; 20 Szentágothai Research Centre University of Pecs Pecs Hungary

**Keywords:** augmented reality, digital learning, distance education, e-learning, medical education, orthopedic training, virtual reality

## Abstract

**Background:**

Digital technologies increasingly shape postgraduate medical education, yet orthopedic and trauma training face unique challenges because of the tactile, procedurally focused skills involved. Digital tools partially address these needs, but gaps remain, particularly across diverse European contexts.

**Objective:**

Our primary aim was to quantitatively assess predictors of digital learning technology acceptance (e-learning, distance education, and virtual reality [VR] and augmented reality [AR]) among European orthopedic and trauma trainees, drawing on the technology acceptance model (TAM) and the unified theory of acceptance and use of technology (UTAUT) as conceptual guides. Specifically, we examined how perceived usefulness and perceived ease of use (TAM), alongside performance expectancy, effort expectancy, social influence, and facilitating conditions (UTAUT), related to trainees’ acceptance of digital technologies. These constructs guided variable selection and grouping, attitudinal scale design, and interpretation of how individual and contextual factors shape acceptance of digital learning tools in orthopedic training. Secondary aims were to describe adoption and attitude patterns, identify attitudinal trainee profiles, and examine contextual associations (eg, workplace type and national gross domestic product [GDP]).

**Methods:**

We distributed a multinational survey via European trainee federations and used validated scales to assess digital competence and attitudes and gathered demographic data (n=217 across 29 European countries). We administered the questionnaire in English; however, respondents who self-reported English proficiency below the intermediate level were excluded from the analyses to minimize potential comprehension-related bias. The survey assessed digital experience, self-reported digital competence, and attitudes toward e-learning, distance education, and VR/AR, and collected detailed demographic and workplace data. Expert review, cognitive pretesting, and pilot testing ensured validity and clarity. Analytical methods included Wilcoxon tests, ANOVA, clustering, multinomial logistic regression, and factor analysis to ensure the reliability and validity of attitudinal measures.

**Results:**

e-Learning technologies were the most widely adopted, whereas VR/AR tools were less frequently used despite high average attitude ratings (mean 4.07, SD 0.88). Cluster analysis identified 3 distinctive groups—enthusiastic, supportive, and hesitant—that differed significantly in digital competence and acceptance profiles. Digital competence and national GDP emerged as significant predictors of group membership, consistent with TAM/UTAUT expectations that perceived capability and contextual facilitating conditions shape acceptance. Variation in attitudes was further associated with workplace type and regional resource disparities, underscoring the influence of contextual factors on technology adoption.

**Conclusions:**

European orthopedic trainees show broad support for digital innovations, preferring VR/AR despite low use. Preliminary evidence supports digital competence as a key mediator of acceptance, with GDP and workplace disparities predicting profiles (hesitant vs enthusiastic). Competence-first strategies and targeted resource equity (eg, low-GDP subsidies), together with policy adjustments, may address regional disparities. Future longitudinal and multimethod studies are needed to test causal pathways implied by TAM and UTAUT and to evaluate the generalizability of these findings across specialties and educational contexts.

## Introduction

In postgraduate medical education, digitalization has progressed in ways that are particularly consequential for procedural specialties [1-4]. Early work in the 1990s established the concept of computer-based surgical simulation and virtual reality (VR) as a training analogue to aviation simulators, creating a foundation for skills rehearsal outside the operating room [1,3-5]. Particularly in surgical fields where technical expertise depends on repeated practice, evidence from the 2000s supported 2 parallel developments: on the one hand, internet-based instruction became an effective modality for knowledge acquisition compared with no intervention and, in many settings, comparable to noninternet formats; and on the other hand, simulation-based education matured into a structured approach for deliberate practice and feedback [6]. For orthopedics, these shifts were not simply “generic” educational upgrades: online platforms enabled scalable delivery of radiograph-based case interpretation, fracture classification modules, and arthroscopy video instruction, while simulation provided a response to reduced hands-on exposure and increasing expectations for patient safety [7,8]. During the 2010s, orthopedic simulation moved beyond low-resolution prototypes toward higher-fidelity VR systems with performance metrics and growing evidence of skill transfer. A multicenter randomized blinded trial demonstrated transfer validity from a VR arthroscopic knee simulator to improved operating-room performance, highlighting that VR can train clinically meaningful technical behaviors [9]. In parallel, haptics-integrated platforms emerged for fixation procedures; for example, a randomized controlled trial on a VR haptics-enabled dynamic hip screw simulator showed measurable training effects without compromising patient safety priorities [10]. Since 2020, the COVID-19 pandemic has accelerated the digital delivery of surgical teaching, while immersive simulation evidence has expanded further, including orthopedic trials showing that adding haptic feedback to immersive VR improves performance and safety metrics in basic bone-drilling tasks [11]. More recently, VR-based simulation has increasingly incorporated automated performance assessment, including artificial intelligence–based approaches, to support scalable feedback and competency tracking [12]. Recent orthopedic-specific syntheses also support VR as an effective adjunct for learning arthroplasty techniques, reinforcing the relevance of acceptance and implementation questions in contemporary training environments [13].

The technological development in the field of medical education has significantly impacted orthopedic and trauma training, where the hands-on nature of the specialty poses unique challenges for digital education [14-16]. In orthopedics and trauma, technical performance is strongly guided by tactile and proprioceptive information from bone–implant interaction and manual reduction maneuvers. For example, fracture reduction requires continuous modulation of traction, rotation, and alignment under variable resistance, while drilling and fixation rely on the operator’s ability to regulate force and torque and recognize changes in bone “feel” to avoid plunge or malposition. In contrast to screen-mediated minimally invasive and robotic procedures, where natural haptic feedback is attenuated, and operators must rely predominantly on visual cues to judge applied force, orthopedic trauma procedures frequently involve direct bone handling and high-load maneuvers in which tactile–proprioceptive information remains central to safe reduction and fixation [17-19]. Accordingly, many digital training solutions (including VR and augmented reality [AR] platforms) still lack robust tactile feedback, which is a critical component in the development of manual skills within surgical disciplines. While some studies report limited measurable benefits of artificial tactile feedback, many users indicate that it enhances the perceived realism and fidelity of simulation environments. Moreover, haptic feedback has been shown to potentially improve specific technical abilities, particularly tasks requiring fine motor control [20]. A recent study showed that absent haptic cues in laparoscopic training caused excessive force and increased tissue damage risk [21]. Digital display variability (eg, monitors, tablets, and head-mounted displays) further impairs visualization and depth perception [20,22]. A 2022 study found haptic-augmented VR superior to nonhaptic VR for orthopedic drilling accuracy, safety, and skills [11]. Most studies concur that haptic feedback is essential for optimal outcomes; advanced systems such as integrated VR/AR simulators or haptic gloves offer promise [23]. Yet user perceptions remain underexplored, with European studies noting heterogeneous validation and uneven hospital integration [24,25]. Cadaveric and supervised exposure still excels for complex orthopedics, necessitating hybrid curricula blending virtual and real-tissue simulation to meet European standards [26].

Despite widespread use, research on trainees’ attitudes toward VR/AR—especially in orthopedics—remains limited, focusing mainly on technical and clinical rather than attitudinal outcomes. While VR/AR shows promise in preoperative planning and simulation, its adoption in postgraduate education, particularly outside the United States, lacks critical evaluation [14,27-29]. The technology acceptance model (TAM) and unified theory of acceptance and use of technology (UTAUT) frameworks address this: TAM emphasizes perceived usefulness (enhanced performance) and perceived ease of use (low effort). UTAUT adds performance expectancy, effort expectancy, social influence, and facilitating conditions (organizational and technical support) [30,31]. In this study, we used the definition of distance education as a structured educational approach characterized by instruction occurring between geographically or temporally separated learners and instructors, using various instructional materials [32,33]. e-Learning), by contrast, refers to teaching and learning activities facilitated through electronic media, encompassing a wide array of devices, software, and digital content [33]. As an umbrella term, e-learning includes diverse methods, with distance education as one application requiring telecommunication. As an umbrella term, e-learning encompasses VR/AR; however, debates persist in simulation-heavy fields such as orthopedics on whether immersive AR/VR qualifies as e-learning or distinct procedural simulation because of its haptic and skill focus—here, we situate VR/AR within e-learning in the context of training following the COVID-19 pandemic [34]. By situating VR/AR within e-learning, this study illustrates how these theoretical models guide understanding of user engagement with cutting‑edge educational tools.

The hybrid TAM–UTAUT model suits orthopedic training: TAM addresses perceived usefulness and ease of use for e-learning, VR, and AR; UTAUT incorporates social influence and facilitating conditions for hierarchical residencies with institutional disparities. Here, digital competence reflects effort expectancy; workplace and gross domestic product (GDP) proxy facilitating conditions [35,36]. Accordingly, the aims of the study were as follows:

- Quantitatively assess acceptance predictors for e-learning, distance education, and VR/AR among European orthopedic trainees, drawing on TAM and UTAUT constructs as conceptual guides for scale selection and interpretation. This addresses limited attitudinal research in procedural specialties beyond technical outcomes.
- Characterize adoption rates and attitude patterns across modalities. Building on low VR/AR use despite high enthusiasm, this maps baseline engagement.
- Identify distinct attitudinal profiles via cluster analysis. Profiles (eg, enthusiastic, supportive, and hesitant) reveal digital competence gradients, aligning with UTAUT effort expectancy.
- Examine associations with contextual factors (workplace type and national GDP). Socioeconomic disparities in digital access—evident in European Union medical training—represent UTAUT facilitating conditions, explaining heterogeneous adoption [30,31].

These aims inform equitable policies for digital integration in diverse European residencies and help ensure that medical device and software development companies effectively integrate digital tools to meet the evolving needs of trainees and institutions in a postpandemic educational landscape.

## Methods

### Ethical Considerations

This study was approved by the Hungarian United Ethical Review Committee for Research in Psychology (reference number 2023-56). Participants provided informed consent at the beginning of the survey. Data were collected anonymously, and no incentives were offered.

### Survey Design and Validation

A self-administered, anonymous online survey was developed, covering demographics, digital competencies, and attitudes toward e-learning, distance education, and VR/AR among orthopedic and trauma trainees, using validated scales (Attitude Toward E-Learning Scale [37], Distance Education Perception Scale [38], and VR/AR attitude items [39]) on a 5-point Likert scale (1=strongly disagree and 5=strongly agree). We did not alter the individual items of the validated scales. Instead, we selected relevant question groups from the original lengthy questionnaire without modifying their wording or content. This approach preserved the integrity and construct validity of the instrument. Expert review, cognitive pretesting, and pilot testing with 10 trainees (qualitative feedback confirmed clarity and comprehensibility) ensured validity and clarity [40]. All items of the survey were presented in English to ensure consistency across countries and to avoid potential discrepancies introduced by translation.

The e-learning and VR/AR-based learning questionnaires demonstrated reliability in our cohort with Cronbach α values of 0.837 and 0.931, respectively. The Distance Education Perception Scale questionnaire consists of 4 subscales: student’s perception, equipment facility, facility and support of the institution, and time management. The first 3 subscales demonstrated high degrees of reliability, ranging from 0.813 to 0.891; however, the time management subscale showed a low level of reliability (α=0.344) and therefore was excluded from the calculations.

Demographics included age, gender, training location (country and city), workplace type (university, regional, city, and rural hospitals), and previous experience with distance education, e-learning, medical simulation, and VR/AR simulation. Digital competence questions covered basic and advanced tools (eg, expertise ranging from basic competencies such as installing a program to advanced skills such as programming or network setup) and VR/AR familiarity (Multimedia Appendix 1).

The structure of the attitudinal scales and analytical framework was informed by the TAM and UTAUT frameworks. In this context, perceived usefulness and ease of use (TAM constructs) were operationalized through items assessing engagement and perceived effectiveness of e-learning, distance education, and VR/AR tools. UTAUT dimensions were represented by social influence and facilitating conditions (eg, equipment availability and workplace resources). These theoretical constructs guided variable grouping, factor structure validation, and subsequent analyses exploring how contextual and personal factors (including GDP, workplace type, and digital competence) influence acceptance and attitudes toward educational technologies.

### Participant Recruitment and Data Collection

Due to the lack of comprehensive registries enumerating orthopedic residents by country, a precise estimation of the eligible population and a predefined sample size were not feasible. Therefore, this study was conducted as a time-bounded, voluntary cross-sectional survey. Participants were recruited via Federation of Orthopaedic and Trauma Trainees in Europe newsletters and mailing lists (estimated reach: approximately 1000-1200 trainees across 27 countries), plus targeted emails and social media posts through national orthopedic resident societies. No incentives offered, reducing response bias from economic and regional disparities. A 2-week data collection period with a 1-week reminder yielded 235 responses (217 analyzed after exclusions). In the absence of centralized registries, an exact response rate was unavailable; however, diverse demographics (29 countries and balanced GDP and workplace types) support generalizability.

The survey was hosted on Google Forms, ensuring ease of access and data security. GDP per capita data were obtained from the International Monetary Fund (2022) and categorized into low (<US $29,000), medium (US $29,000-US $40,000), and high (>US $40,000) GDP groups (tertiles of 29 respondent countries), aligning with World Bank thresholds (the European Union average is US $41,806).

### Statistical Analysis

To evaluate trainees’ attitudes toward e-learning and distance education, mean scores for survey items were compared with the neutral midpoint (3.0) using Wilcoxon signed-rank tests due to nonnormal distributions indicated by Shapiro-Wilk tests (all *P*<.01). Effect sizes were reported as rank-biserial correlations (r_bs_), with values of 0.10, 0.30, and 0.50 interpreted as small, medium, and large effects, respectively. To examine whether attitudes differed depending on the type of workplace and the economic context of the country, separate 2-way ANOVAs were run with workplace type (university vs nonuniversity clinics) and GDP per capita (low, medium, or high) as between-subject factors, and attitude variables (ie, scores on subscales of the attitude questionnaires) as outcomes. Significant interactions were followed up by simple effect analyses. All post hoc analyses were adjusted for multiple testing using the Bonferroni correction.

A priori power analyses using G*Power (version 3.1.9.7 [41]) indicated that the minimum sample size required for ANOVA was 155 (α=5%; power=80%), and thus the final sample of 217 (see below) had adequate statistical power to detect main effects as well as the interactions examined. Differences in experience levels and digital competencies across domains were tested using the Friedman test. Significant results were followed up by Wilcoxon tests with Bonferroni correction. Spearman partial correlations were used to assess relationships among the measures while controlling for digital competence and experience with digital learning. When an attitude variable was correlated with either competence or experience, the other served as the control variable. No control variables were applied for the correlation between competence and experience. Spearman correlations were chosen because Shapiro-Wilk tests indicated nonnormal distributions for all variables (all *P*<.05), except for the student’s perception subscale.

We used k-means cluster analysis to identify distinct subgroups of participants with similar attitudinal profiles toward digital learning. The optimal cluster count was determined using the elbow and silhouette methods (Multimedia Appendix 2). Cluster-wise stability was assessed using the mean Jaccard similarity value obtained via bootstrapping (n=1000). Mean Jaccard similarity values >0.75 and >0.85 were interpreted as indicators of stable or very stable clusters, respectively [42].

After clustering, the dataset was aggregated to calculate mean values for each cluster, providing insight into the dominant attitudes and experiences regarding digital learning across groups. Multinomial logistic regression was conducted to examine how background variables (eg, digital competence, experience, and demographics) predicted cluster membership and to better understand differences between subgroups.

To identify the underlying factor structure and consolidate conceptually overlapping constructs into a clearer and more parsimonious model, exploratory factor analysis (EFA) was conducted on the complete set of items from all 3 attitude questionnaires. Because items were ordinal, a polychoric correlation matrix was computed, and robust maximum-likelihood extraction with oblique (Promax) rotation was used. Factor retention was guided by (1) parallel analysis and scree plot inspection and (2) interpretability; primary loadings ≥0.40, cross-loadings <0.30, and communalities ≥0.30 were used as criteria for item retention. The full EFA diagnostics, including the parallel-analysis scree plot, communalities, and item loadings, are provided in Multimedia Appendix 3.

To confirm the factor structure identified by EFA, confirmatory factor analysis was conducted using robust maximum likelihood estimation (estimator=“MLR”) to account for nonnormality. Model adequacy was evaluated using standard fit indices and recommended thresholds: comparative fit index (CFI) and Tucker-Lewis index (TLI) ≥0.90 (acceptable) or ≥0.95 (excellent), root mean square error of approximation (RMSEA) ≤0.08 (acceptable) or ≤0.06 (good), and standardized root mean square residual (SRMR) ≤0.08. Standardized factor loadings ≥0.50 were interpreted as adequate. Model refinement involved reviewing modification indices (MI>10) and applying theoretically justified correlations between residuals where appropriate.

Statistical significance was set at *P*<.05 (2-tailed) for all analyses unless Bonferroni-corrected otherwise. All analyses were conducted using SPSS (version 28; IBM Corp) and R (version 4.4.0; R Foundation for Statistical Computing). R packages used are listed in Multimedia Appendix 4.

## Results

### Overview

In total, 235 responses from 29 countries (geographical distribution shown in Multimedia Appendix 5) were received, of which 217 were included in the analysis after excluding responses with beginner-level English proficiency (n=17) or incomplete data (n=1) to minimize potential bias arising from language-related comprehension issues. Excluded respondents did not significantly differ from included respondents in terms of basic demographics (age: *U*=1482.5; *P*=.17; r_bs_=0.2 and gender: *χ*^2^_1_=0.02; *P*=.88); however, trainees working at university clinics were underrepresented among the excluded participants (*χ*^2^_1_=5.3, *P*=.02). A detailed description is provided in Table 1. The dataset is available elsewhere [43].

**Table 1:** Participant demographics and background characteristics.

Characteristics		Values
Sample size, n		217
Age (years), mean (SD); range		31.5 (4.38); 24-45
Training duration (years), mean (SD); range		4.31 (2.20); 1-7
**Gender, n (%)**		
	Male	156 (71.9)
	Female	61 (28.1)
**English proficiency, n (%)**		
	Intermediate	95 (43.7)
	Professional	111 (51.2)
	Mother tongue	11 (5.1)
**Workplace type, n (%)**		
	University clinical center or national referral hospital	116 (53.5)
	Regional/provincial or county referral hospital	46 (21.2)
	City hospital	37 (17.1)
	Town or rural hospital	18 (8.3)
**GDP^a^** **per capita group (US $), n (%)**		
	Low (< 29,000)	88 (40.6)
	Medium (29,000-40,000)	69 (31.8)
	High (>40,000)	60 (27.6)

^a^GDP: gross domestic product.

### Experience and Competence Levels

Participants reported their experience across 4 domains: distance education, e-learning, medical simulation, and VR/AR. The Friedman test indicated significant differences in self-reported experience across domains (*χ*^2^_3_=285.0; *P*<.001). Post hoc Wilcoxon signed-rank tests indicated that participants’ experience with e-learning was significantly higher than their experience with distance education (Wilcoxon Z=3.68; *P*=.001; r_bs_=0.44), medical simulation (Z=9.30; *P*<.001; r_bs_=0.88), and VR/AR (Z=11.12; *P*<.001; r_bs_=0.97). Experience with distance education was also significantly higher than experience with medical simulation (Z=6.62; *P*<.001; r_bs_=0.61) and VR/AR (Z=9.88; *P*<.001; r_bs_=0.86). Finally, experience with medical simulation was significantly higher than that with VR/AR (Z=8.34; *P*<.001; r_bs_=0.88).

The Friedman test showed significant differences in self-reported digital competence (*χ*^2^_4_=542.9; *P*<.001). Post hoc analyses revealed that competence in basic tools and online platforms did not differ significantly (Z=2.76; *P*=.06; r_bs_=0.31), but both were rated significantly higher than each of the other domains of competence (all *z*>10.51, all *P*<.001, and all effect sizes large per Cohen r_bs_ > 0.97). Nearly 80% of participants reported “advanced” or “expert” competence in both domains. Competence in digital problem-solving was rated significantly higher than both competence in advanced technologies (Z=5.55; *P*<.001; r_bs_=0.55) and VR/AR (Z=9.60; *P*<.001; r_bs_=0.88). Finally, self-reported competence in advanced technologies was significantly higher than that of VR/AR (Z=7.20; *P*<.001; r_bs_=0.70). For digital problem-solving, advanced technologies, and VR/AR, 37.79% (82/217), 24.88% (54/217), and 11.06% (24/217) reported “advanced” or “expert” level of competence, respectively. Multimedia Appendix 6 provides a more detailed description of within-domain distributions.

### Attitudes Toward e-Learning and Distance Education

**Figure 1:**
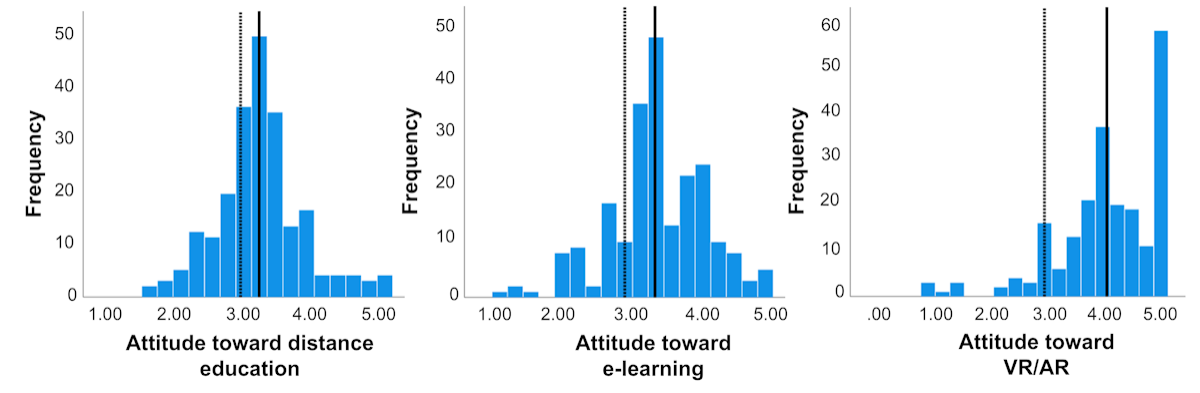
Histograms of the mean scores on the attitude questionnaires. The dashed line indicates the neutral midpoint (3.00), whereas the solid line indicates the sample mean. AR: augmented reality; e-learning: electronic learning; VR: virtual reality.

Attitudes toward e-Learning and distance education varied among participants. Distance education recorded a mean score of 3.27 (SD 0.63) and a median of 3.18 (IQR 2.95-3.55), indicating moderately positive attitudes, with consistent responses (SD 0.63; skewness 0.37). Statistical analysis revealed significant positivity in the attitudes toward distance education (Wilcoxon Z=6.10; *P*<.001; r_bs_=0.49; mean 3.27 (SD 0.63); 95% CI 3.19-3.36). e-Learning followed with a mean of 3.43 (SD 0.69) and a median of 3.44 (IQR 3.11-3.89), exhibiting slightly more favorable attitudes with moderate variability (SD 0.69). The Wilcoxon test confirmed the positivity of these attitudes (Z=8.03; *P*<.001; r_bs_=0.64; mean 3.43 (SD 0.69); 95% CI 3.33-3.52). VR/AR received the highest mean score of 4.07 (SD 0.88) and a mode of 5, reflecting the most positive attitudes, albeit with greater variability (SD 0.88, skewness –1.17), and the analysis significantly supported these positive perceptions (Z=10.94; *P*<.001; r_bs_=0.89; mean 4.07 (SD 0.88); 95% CI 3.96-4.19). Negative skewness in VR/AR attitudes suggests a ceiling effect, potentially reflecting aspirational responding. Overall, VR/AR elicited the strongest positive responses, although its variability suggested mixed familiarity or application, whereas distance education and e-learning garnered more tempered but consistent favorability (Figure 1).

### Cluster Analysis

**Figure 2:**
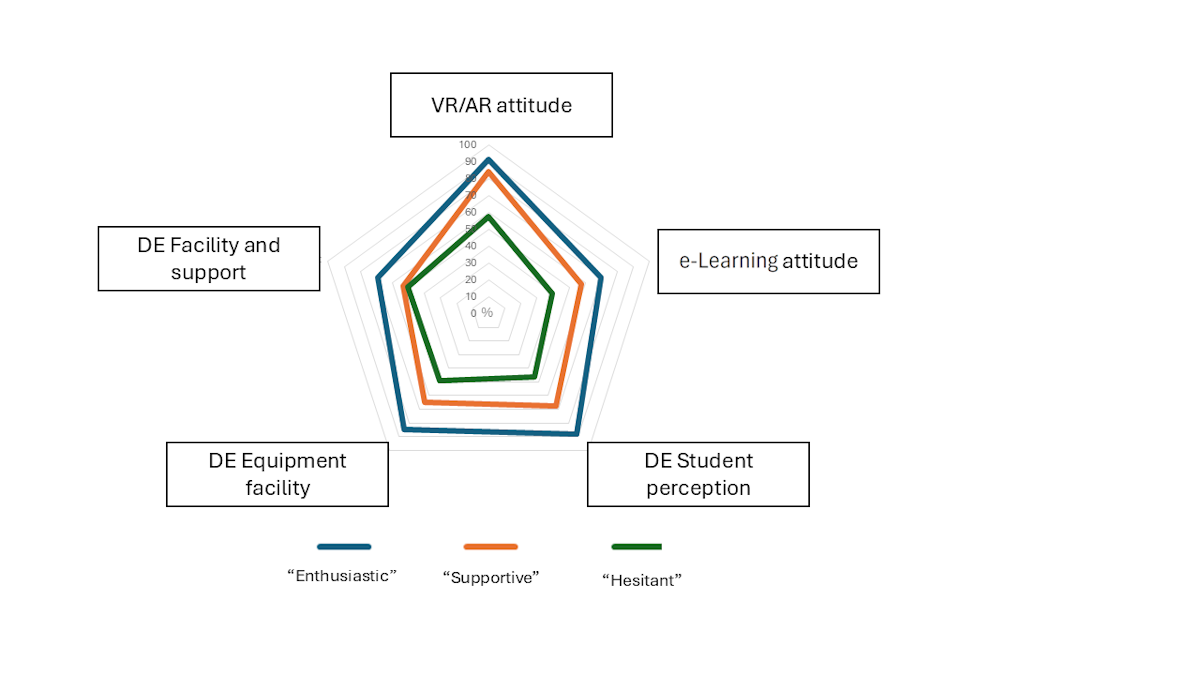
Radar chart illustrating the distribution of standardized (0%-100%) mean scores across 3 clusters: “enthusiastic,” “supportive,” and “hesitant.” Scores were normalized based on each dimension’s theoretical maximum to ensure comparability across variables. The 5 dimensions include attitude toward VR/AR, attitude toward e-learning, distance education (DE) student’s perception, DE equipment facility, and DE facility and support.

K-means clustering based on responses to the attitude scales, optimized for internal coherence and separation, suggested 3 clusters that provided an optimal balance of granularity and distinctiveness. The average silhouette score was 0.27 for the 3-cluster solution, whereas it was 0.26 and 0.21 for the 2- and 4-cluster solutions, respectively (Multimedia Appendix 2 provides a detailed description of cluster number optimization). The clusters were tentatively named “enthusiastic” (47/217, 21.66%), “supportive” (129/217, 59.45%), and “hesitant” (41/217, 18.89%) (Figure 2). All 3 clusters showed high levels of stability according to mean Jaccard similarities (0.85, 0.86, and 0.89), confirming the robustness of the 3-cluster solution (mean Jaccard similarities for the 2-cluster solution: 0.87 and 0.88; for the 4-cluster solution: 0.57, 0.30, 0.57, and 0.74). The attitudes were measured using scales adapted from validated sources, including the Attitude Toward E-Learning Scale, the Distance Education Perception Scale, and items related to perceptions of VR/AR applications in learning. Enthusiastic respondents showed the highest endorsements across all attitude scales. Supportive individuals expressed favorable attitudes but with moderate enthusiasm compared with the enthusiastic cluster. Hesitant respondents exhibited the lowest agreement with positive statements regarding all educational technology aspects (Figure 2). Significant differences among clusters were confirmed by 1-way ANOVAs across all attitude dimensions, each showing large effect sizes (η_p_^2^>0.14): distance education student’s perception, *F*_2,214_=179.85; *P*<.001; η_p_^2^=0.63; distance education equipment facility, *F*_2,214_=132.94; *P*<.001; η_p_^2^=0.55; distance education facility and support, *F*_2,214_=52.27; *P*<.001; η_p_^2^=0.33; e-learning engagement, *F*_2,214_=105.04; *P*<.001; η_p_^2^=0.50; and VR/AR engagement, *F*_2,214_=50.77; *P*<.001; η_p_^2^=0.32.

The results of multinomial logistic regression analysis are summarized in Table 2. Global model fit indices indicated that the multinomial regression model provided a significantly better fit than the null model (*χ*^2^_14_=45.27; *P*<.001) with a McFadden pseudo-*R*^2^ of 0.11, suggesting a modest yet meaningful amount of variance in cluster membership was accounted for by the predictors. The findings highlighted digital competence as a significant predictor in differentiating between the enthusiastic and hesitant clusters (estimate=0.22; SE=0.07; Z=3.22; *P*=.001; and odds ratio [OR] 1.25, 95% CI 1.09-1.43) and between the supportive and hesitant clusters (estimate=0.18; SE=0.06; Z=3.07; *P*=.002; OR 1.19, 95% CI 1.07-1.33). This indicates that higher levels of digital competence are associated with increased odds of belonging to the enthusiastic or supportive clusters rather than the hesitant cluster, suggesting that perceived capability with digital technologies plays a key role in shaping trainees’ attitudinal profiles. Economic conditions, indicated by GDP, also played a significant role in distinguishing between the enthusiastic and hesitant groups (estimate=–0.03; SE=0.01; Z=–2.44; *P*=.02; OR 0.97, 95% CI 0.95-0.99) Similarly, GDP was a significant predictor of cluster membership when the enthusiastic and supportive groups were compared (estimate=–0.03; SE=0.01; Z=–3.02; *P*=.003; OR 0.97, 95% CI 0.95-0.99), suggesting that trainees from countries with lower GDP were more likely to belong to the enthusiastic cluster. Gender, age, training year, workplace type (university vs nonuniversity clinic), and prior digital experience were not significant predictors (all *P*>.05). Given that demographic and training characteristics, as well as overall experience with various forms of digital learning, did not strongly differentiate clusters, attitudinal patterns may be largely independent of these background factors.

**Table 2:** Multinomial regression analysis comparing attitudinal clusters in distance education.

Clusters and predictors					Estimate			SE			Wilcoxon Z		*P* value			OR^a^ (95% CI)
**Enthusiastic** **(47/** **217** **, 21.66%)** **–hesitant** **(41/** **217** **, 18.89%)**																
	Intercept			-5.07			2.35			-2.16		.03			0.01 (0.00-0.63)	
	Digital_experience			0.1			0.07			1.38		.17			1.11 (0.96-1.28)	
	Digital_competence^b^			0.22			0.07			3.22		.001			1.25 (1.09-1.43)	
	Training_year			0.09			0.14			0.65		.52			1.1 (0.83-1.46)	
	**Sex**															
		Male–female	0.5			0.58			0.86			.39		1.65 (0.52-5.19)		
	**Workplace**															
		University clinic– nonuniversity clinic	-0.6			0.48			-1.24			.21		0.55 (0.21-1.41)		
	GDP^c^ (US $1000)^b^			-0.03			0.01			-2.44		.02			0.97 (0.95-0.99)	
	Age (years)			0.03			0.07			0.38		.70			1.03 (0.89-1.19)	
**Supportive** **(129/** **217** **, 59.45%)** **–hesitant** **(41/** **217** **, 18.89%)**																
	Intercept			-0.45			1.9			-0.24		.81			0.64 (0.02-26.52)	
	Digital_experience			0			0.06			0.02		.99			1 (0.89-1.12)	
	Digital_competence^b^			0.18			0.06			3.07		.002			1.19 (1.07-1.33)	
	Training_year			0.11			0.12			0.93		.35			1.11 (0.89-1.39)	
	**Sex**															
		Male–female	0			0.42			0.01			.99		1 (0.44-2.29)		
	**Workplace**															
		University clinic–nonuniversity clinic	-0.39			0.39			-1.01			.31		0.67 (0.31-1.45)		
	GDP_(US $1000)			0			0.01			0.04		.97			1 (0.98-1.02)	
	Age (years)			-0.04			0.06			-0.66		.51			0.96 (0.85-1.08)	
**Enthusiastic** **(47/** **217** **, 21.66%)** **–supportive** **(129/** **217** **, 59.45%)**																
	Intercept			-4.62			1.78			-2.60		.009			0.01 (0.00-0.32)	
	Digital_experience			0.10			0.06			1.75		.08			1.11 (0.99-1.24)	
	Digital_competence			0.05			0.05			0.94		.35			1.05 (0.95-1.16)	
	Training_year			-0.01			0.11			-0.12		.91			0.99 (0.79-1.23)	
	**Sex**															
		Male–female	0.50			0.49			1.02			.31		1.64 (0.63-4.28)		
	**Workplace**															
		University clinic–nonuniversity clinic	-0.21			0.37			-0.56			.58		0.81 (0.39-1.69)		
	GDP (US $1000)^b^			-0.03			0.01			-3.02		.003			0.97 (0.95-0.99)	
	Age (years)			0.07			0.06			1.23		.22			1.07 (0.96-1.19)	

^a^OR: odds ratio.

^b^Denotes statistically significant values.

^c^GDP: gross domestic product.

### Attitude as a Function of Workplace and GDP of the Country Hosting the Training Program

**Figure 3:**
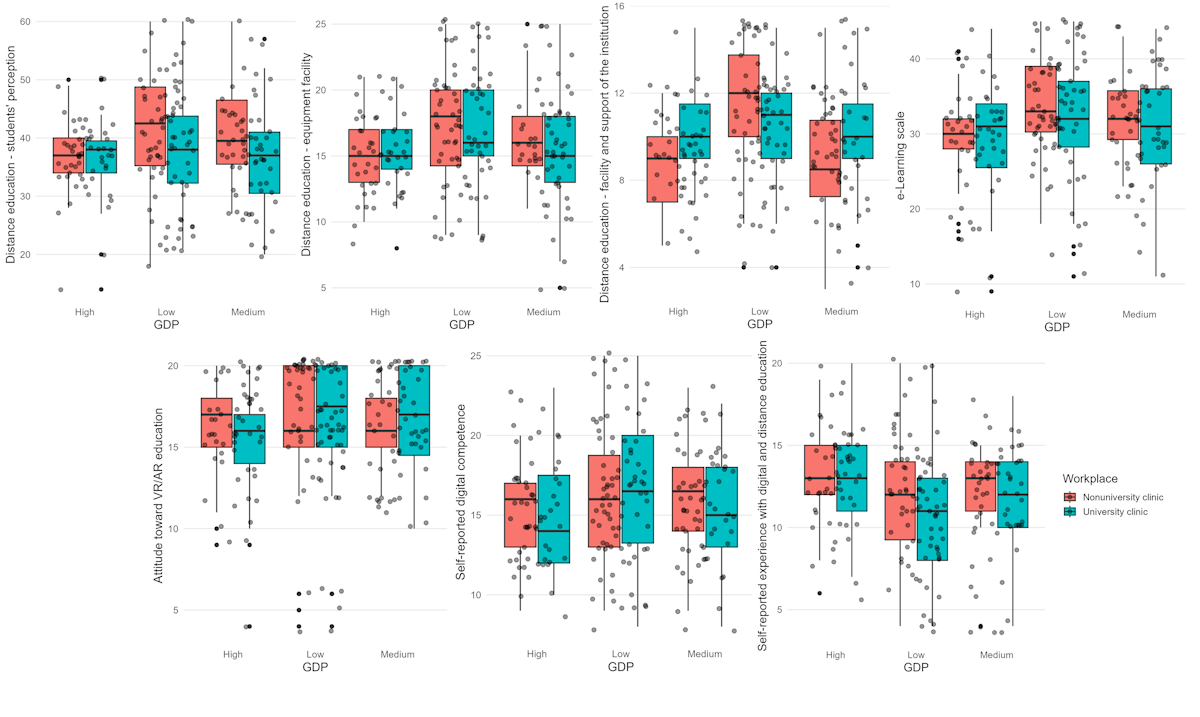
Results of the questionnaire scores as a function of workplace and gross domestic product (GDP) of the country hosting the training program.

The results of the ANOVAs investigating the effects of GDP and workplace on attitudes are depicted in Figure 3 and Multimedia Appendix 7. The main effect of workplace reached significance for the student’s perception subscale (*F*_1,211_=6.95; *P*=.009; η_p_^2^=0.03). Trainees from nonuniversity clinics had significantly more positive attitudes toward distance learning than trainees from university clinics.

The analysis of the equipment facility subscale yielded a significant GDP main effect (*F*_2,211_=5.02; *P*=.007; η_p_^2^=0.05). Corrected post hoc tests showed that the scores of trainees from low-GDP countries were significantly higher than those of trainees from high-GDP countries (mean difference –2.07, 95% CI –3.64 to –0.49; t_211_=–3.10; 2-tailed *P*=.007); Cohen *d*=–0.52). Similarly, there was a significant GDP main effect for facility and support of the institution (*F*_2,211_=7.84; *P*<.001; η_p_^2^=0.07), as well as a significant Workplace × GDP interaction (*F*_2,211_=5.16; *P*=.007; η_p_^2^=0.05). Simple effects analyses revealed that the GDP main effect was only significant for trainees from nonuniversity clinics (*F*_2,211_=13.24; *P*<.001; η_p_^2^=0.11). Within this group, scores of trainees from low-GDP countries were significantly higher than those of trainees from medium-GDP (mean difference 2.17, 95% CI 0.33 to 4.01; t_211_=3.39; 2-tailed *P*=.01); Cohen *d*=–1.11) and high-GDP countries (mean difference –2.73, 95% CI –4.35 to –1.11; t_211_=–4.85; 2-tailed *P*<.001; Cohen *d*=–1.11). The analyses also showed a significant workplace effect within the group of trainees from high-GDP countries (*F*_1,211_=6.03; *P*=.02; η_p_^2^=0.03): scores were higher for trainees from university clinics than for other trainees.

The analysis of attitudes toward e-learning also yielded a significant GDP main effect (*F*_2,211_=4.59; *P*=.01; η_p_^2^=0.04): trainees from low-GDP countries showed significantly more positive attitude toward e-learning than trainees from high-GDP countries (mean difference –3.62, 95% CI –6.45 to –0.80; t_211_=–3.03; 2-tailed *P*=.008; Cohen *d*=–0.51).

Finally, a significant GDP main effect was found for experience with technologies (*F*_2,211_=3.65; *P*=.03; η_p_^2^=0.03): trainees from high-GDP countries had significantly more experience than trainees from low-GDP countries (mean difference 1.48, 95% CI 0.14 to 2.82; t_211_=2.60; 2-tailed *P*=.03; Cohen *d*=0.44). No further significant main effects or interactions were found.

### Associations Among Digital Competence, Experience, and Attitude

**Figure 4:**
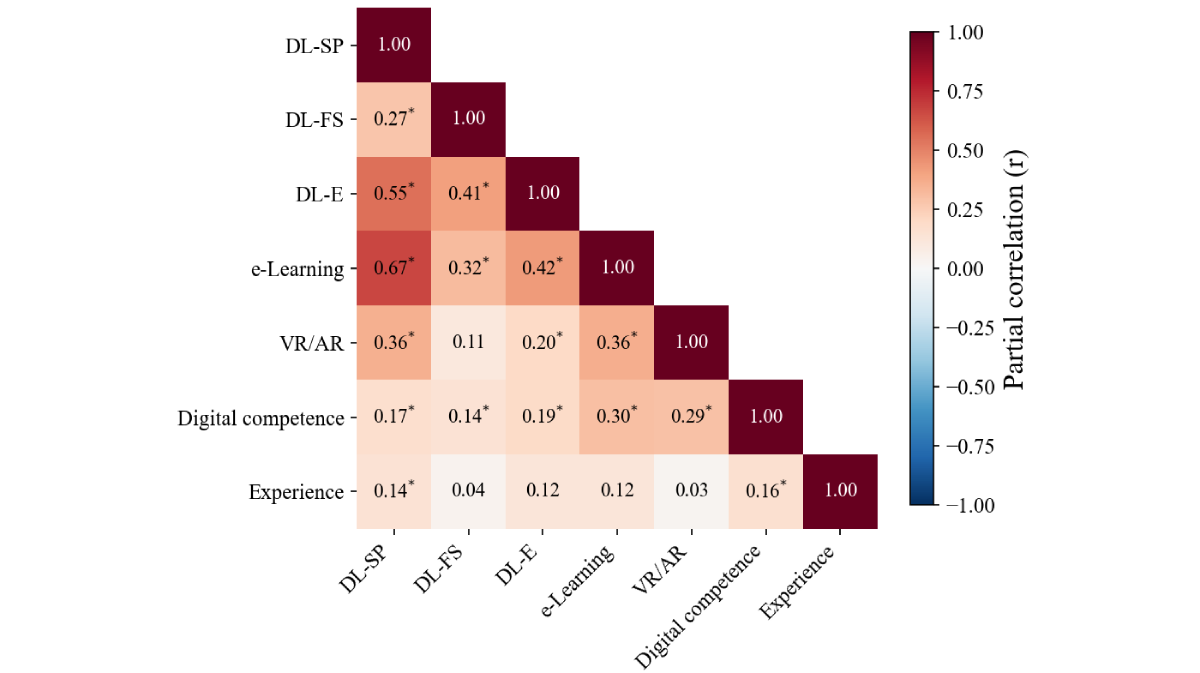
Correlations and partial correlations between attitude scales, digital competence, and experience with digital technology. Control variables were digital competence and experience with digital learning. When competence or experience was correlated with an attitude variable, the other variable served as the control variable. No control variables were applied when examining the correlation between competence and experience. *Indicates statistical significance (*P*<.05). DL-E: distance learning–equipment facility; DL-FS: distance learning–facility and support of the institution; DL-SP: distance learning–students’ perceptions; e-Learning: electronic learning; VR/AR: virtual reality/augmented reality.

Correlation analyses (Figure 4) showed digital competence was strongly associated with favorable attitudes across all learning modalities, especially in low- and medium-GDP countries (Multimedia Appendix 8). Prior experience also correlated with more positive attitudes, highlighting the role of familiarity. Positive views of one modality often extended to others, reflecting broad openness to digital education, consistent with theoretical frameworks emphasizing usability and prior exposure.

### Factor Analysis of Attitude Items

The Mardia test showed significant multivariate skewness (b_1p_=307.11; *P*<.001) and kurtosis (b_2p_=1379.52; *P*<.001), indicating violation of multivariate normality. Consequently, a polychoric correlation matrix was used in all subsequent analyses. Factorability was supported by the Bartlett test of sphericity (*χ*^2^_595_=7216.854; *P*<.001), the Kaiser-Meyer-Olkin index =0.78, and the determinant of the correlation matrix (3.97×10^–16^). Interitem correlations were generally moderate (median rₛ=0.368, range 0-0.925). Parallel analysis indicated 6 factors; however, conceptual overlap among related dimensions motivated a theory-driven consolidation (eg, distance education engagement and conditions → distance education perception), yielding a more parsimonious 4-factor solution. In this reduced model, after rotation, the 4 factors explained 20.8%, 14.2%, 11.8%, and 10.7% of the variance (total=57.5%). No Heywood cases were identified. More information is available in Multimedia Appendix 3.

For model refinement, items with strong primary loadings and adequate communalities were retained, while theoretical interpretability was also considered. Further, 8 items (e-learning items 6 and 10; distance education items 7, 10, 12, and 20-22) were removed because of low communalities. Distance education item 8 was excluded because of a low loading (0.38) and cross-loading. e-Learning items 7-9 and 11 were excluded because they loaded on a theoretically implausible factor (distance education perception rather than e-learning).

The resulting model comprised 4 latent constructs: distance education perception, equipment facility perception, attitude toward VR/AR, and attitude toward e-learning. This structure was evaluated using confirmatory factor analysis. The initial model showed acceptable fit (*χ*^2^_203_=370.57; CFI=0.938; TLI=0.93; RMSEA=0.062; SRMR=0.052), although modification indices indicated potential improvement. Accordingly, a residual covariance was specified between distance education items 13 and 14 (MI=33.49), resulting in improved fit (ΔCFI=0.01; ΔTLI=0.011; ΔRMSEA=–0.005; ΔSRMR=0.002). Subsequent indices suggested an additional residual covariance between e-learning items 1 and 2 (MI=24.52). In both cases, the items were semantically similar and reflected closely related constructs, justifying correlated residuals (Multimedia Appendix 3).

**Figure 5:**
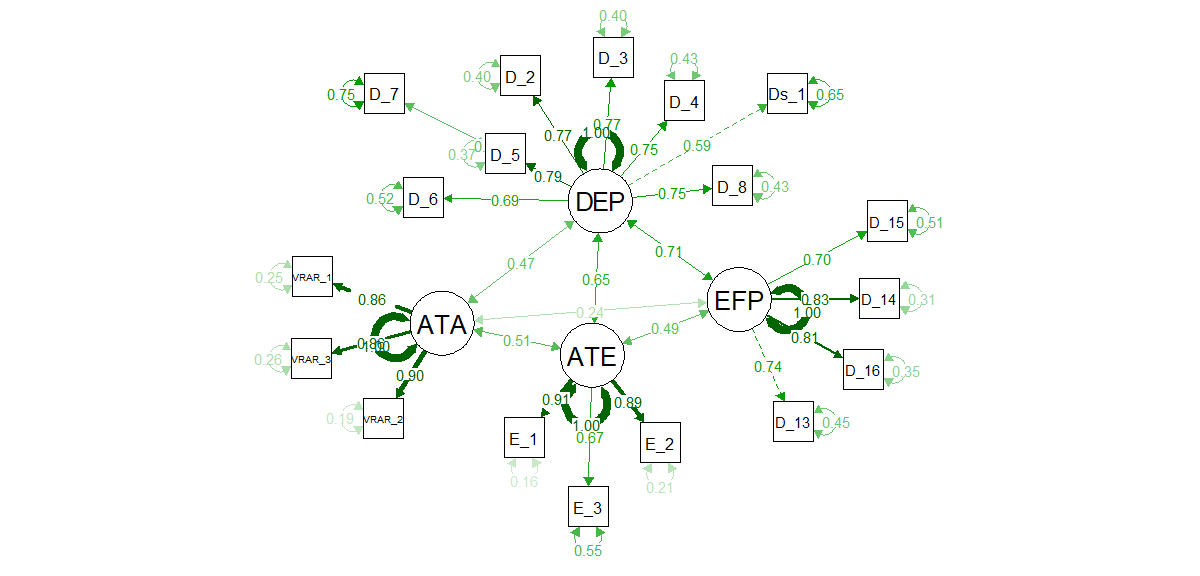
Interfactor correlations among constructs in the confirmatory factor analysis model. ATA: attitude toward VR/AR; ATE: attitude toward e-learning; DEP: distance education perception; EFP: equipment facility perception.

The final model (Figure 5) demonstrated good fit (*χ*^2^_201_=323.203; CFI=0.955; TLI=0.948; RMSEA=0.053; SRMR=0.053). Further modifications were not pursued despite several MIs>10 to avoid overfitting. The model showed moderate to high correlations among the constructs, reflecting their conceptual interdependencies within the domain of e-learning environments. In more detail, interfactor correlations demonstrated meaningful relationships, with distance education perception positively associated with equipment facility perception, attitude toward VR/AR, and attitude toward e-learning, and equipment facility perception significantly correlated with both attitude toward VR/AR and attitude toward e-learning, reflecting theoretical interdependencies across educational dimensions. Findings from this more parsimonious model were consistent with the partial correlation patterns reported earlier, supporting the stability of the factor structure.

## Discussion

### Overview

Digital tools have advanced medical education, particularly in procedural fields such as orthopedics, where tactile skills challenge remote learning. VR/AR simulations enabling safe rehearsal (eg, arthroscopy knee simulators show operating room transferability, and hip arthroscopy VR matches benchtop training) [44-46]. This study highlights the importance of digital competence and familiarity in shaping attitudes, aligning with findings from other disciplines such as nursing and parallel surgical evidence from veterinary medicine [47-50].

Our finding that VR/AR elicited the most positive attitudes (mean 4.07) despite limited exposure aligns with recent umbrella reviews showing VR’s growing dominance in surgical training and procedural skill acquisition [34]. The findings of Sommer et al [51] regarding VR’s reduction of errors and improvement of completion times in surgical simulations (60 students; 2-arm randomized trial) are consistent with the trainee optimism reported in this study. This is corroborated by the findings from Pettinelli et al [13], whose systematic review with a total sample size of 148 residents or students affirmed that VR is effective for learning specific arthroplasty techniques (no difference between VR-trained and control groups; *P*=.56; *I*^2^=85%), further supporting the notion that residents are open to integrating this technology into their training repertoire. However, similar to the narrative review by Ivanov et al [52], we note that statistical significance in performance outcomes remains inconsistent, underscoring the need for larger longitudinal studies.

Structural equation modeling (SEM) revealed factors that influence clinical cognition, health behavior, or the acceptance of digital learning modalities [53-55]. Our analysis demonstrates that engagement with distance education shows a strong association with acceptance, suggesting a bidirectional influence between perceptions and intent in shaping residents’ comfort with e-learning platforms and VR/AR tools. This finding underscores the importance of fostering engagement through distance education to build familiarity with advanced digital tools. SEM associations (eg, distance education acceptance) are correlational, precluding causality; bidirectionality is likely (eg, comfort shapes perceptions and vice versa). Longitudinal studies are needed to test directions and moderators such as competence.

Our SEM findings, which link digital confidence to VR/AR acceptance, support the “blended digital comfort” strategies described by Cabero-Almenara et al [56] (online questionnaire involving 17,301 Latin American students). For example, pairing VR modules with hands-on workshops could reduce effort expectancy (a UTAUT construct), as observed in proof-of-concept mixed-reality ward rounds by Bala et al [57] involving 11 students, in which 82% of trainees reported improved interaction. Tool proficiency is key for acceptance and integration.

The interplay between e-learning dimensions, such as blended digital comfort’s influence on e-learning attitudes, emphasizes the need to address broader digital literacy and confidence. Consistent with prior research (multinational survey involving 223 students), positive attitudes were more pronounced in settings with lower access to conventional resources, such as noncentral hospitals and low-GDP countries, where distance and e-learning provide critical educational support [58]. This is particularly important for designing interventions that integrate multiple technologies effectively.

Beyond economic resources, sociocultural factors also shape regional differences in technology adoption; national cultural dimensions (uncertainty avoidance, individualism/collectivism, and power distance) moderate links among ease of use, perceived usefulness, and intention to adopt [59]. Trainees from lower-GDP countries showed more positive attitudes, possibly reflecting adaptive innovation cultures rather than mere scarcity, whereas high-GDP regions may have more rigid educational norms and accreditation structures that slow adoption (Multimedia Appendix 9 presents the Hofstede dimensions of countries included in this study) [60]. Effective VR-based orthopedic education therefore requires strategies tailored to both economic capacity and cultural context: in lower-income settings, low-cost standalone VR, local VR “champions,” and team-based modules can support uptake, whereas in higher-income settings, integration into accredited curricula, assessment frameworks, and faculty development programs helps overcome inertia. Across contexts, culturally responsive leadership, clear standards, and evaluation using frameworks such as the Consolidated Framework for Implementation Research and reach, effectiveness, adoption, implementation, and maintenance (RE-AIM) are needed to sustain engagement, equity, and long-term integration [61,62].

TAM and UTAUT provide a useful interpretive lens for our attitudinal patterns, linking them to concepts such as perceived usefulness, ease of use, and contextual enablers. In our model, attitude toward e-learning and distance education perception map onto perceived usefulness and performance expectancy, with digital competence and experience reflecting effort expectancy, and workplace type and GDP representing facilitating conditions and social influence. Digital competence showed positive associations with attitudes toward e-learning, distance education, and VR/AR—patterns consistent with TAM and UTAUT concepts, while institutional effects align with UTAUT’s emphasis on environmental support and broader trends in which novel tools elicit optimism despite limited familiarity, although we did not estimate a full structural model (eg, with *R*^2^ for acceptance) [63,64]. Perceived ease of use, digital confidence, and facilitating conditions—access to hardware, connectivity, and institutional backing—are therefore critical and can be strengthened through user-friendly tools, targeted training, institutional support, subsidized technology, and partnerships with providers to promote equitable access [65].

### Implications for Educational Design

The findings suggest a tiered approach to educational design. At the core (immediate) level, digital competence training should be embedded within curricula through targeted workshops and organized testing campaigns to enhance familiarity and effort expectancy. At the supportive (medium-term) level, access to digital tools should be subsidized to address disparities associated with lower GDP and strengthen facilitating conditions. Collaborative programs and partnerships between institutions in lower- and higher-GDP countries should also be promoted to support resident training, faculty development, and the sharing of educational resources and programs. At the advanced (long-term) level, VR/AR modules and e-learning platforms can be integrated into hybrid educational models, with content and implementation strategies tailored to local cultural and contextual needs.

### Limitations

Limitations include the unknown size of the trainee population, uneven geographic distribution, inability to characterize nonrespondents, and reliance on self-reported data. Due to the absence of standardized, publicly available databases enumerating orthopedic resident characteristics across European countries, it is not possible to ascertain whether the proportions observed in our sample reflect the true population. Uneven geographic representation limits the ability to extrapolate findings uniformly across all European contexts. Exclusion bias might have affected the results because of the higher exclusion rate among trainees working in nonuniversity clinics (10 of 18 exclusions were due to beginner-level English proficiency). The reliance on self-reported attitude data may have introduced social desirability bias, potentially inflating positive responses toward digital tools, as evidenced by the VR ceiling. No social desirability scale was used. Nonetheless, the findings are likely generalizable because diverse demographics (29 countries and balanced GDP and workplace types) support generalizability, as do global similarities in medical training and digital education challenges. Future multimethod validation (eg, objective logs) is needed; longitudinal studies are needed to test causality.

### Future Perspectives

Further studies should assess the effects of sustained digital platform exposure on attitudes and outcomes over time, using mixed methods designs. Qualitative components should examine VR/AR as innovative tools for orthopedic and trauma trainee education. Phenomenological studies can explore trainees’ lived experiences with VR/AR tools, while institutional case studies can reveal local implementation mechanisms. Quantitative designs (exploratory, experimental, and longitudinal) should assess skills acquisition via the objective structured assessment of technical skills and the objective structured clinical examination across residency. Translational research should examine the impact of digital skills on patient outcomes and their correlations with attitudes, motivation, and retention. All studies should be grounded in self-directed learning, cognitive load, and deliberate practice theories for design and interpretation.

Based on the findings, future research should focus on 3 main directions:

- Longitudinal randomized controlled trials (priority): track TAM and UTAUT pathways over time; test interventions (eg, competence training) on actual method adoption in multisite trials (n>500).
- Qualitative studies: phenomenology or interviews elucidate mechanisms (eg, barriers among hesitant trainees and drivers of enthusiasm in low-GDP countries).
- Mixed methods evaluations: assess policy impacts (eg, subsidies and curricula) via surveys and usage logs in hybrid programs across Europe’s diverse socioeconomic landscape.

To operationalize these research directions and secure funding, we propose a scalable 4-step approach (Textbox 1) aligned with European Union schemes (Erasmus+ and Horizon Europe) and frameworks (Consolidated Framework for Implementation Research for determinants; RE-AIM, and dynamic sustainability framework for sustainability). Each includes milestones, risks, and mitigations (more detailed suggestions are provided in Multimedia Appendix 10).

**Scalable 4-step implementation and funding roadmap for virtual reality/augmented reality (VR/AR) surgical training in the European Union.**

**Step 1 (year 1)**

Generate feasibility evidence via pilot studies in 3 to 5 centers (approximately 30-50 trainees). End points: recruitment rates, usability, and faculty burden. Fund curriculum and cross-border activities via Erasmus+ Small-scale Partnerships; mitigate heterogeneous infrastructure with device specifications and train-the-trainer programs.

**Step 2 (years 2-3)**

Conduct preregistered multicenter studies (n≥150 trainees) with standardized outcomes (objective structured assessment of technical skills and the Surgical Council on Resident Education), efficiency analyses, and cost-benefit analyses. Compare virtual reality (VR) and augmented reality (AR) with traditional methods; mitigate bias via cluster designs and harmonized training.

**Step 3 (years 3-4)**

Establish 2 to 4 regional VR and AR hubs for training, content sharing, and faculty development, including low-resource sites. Leverage the Society in Europe for Simulation Applied to Medicine and European Digital Innovation Hubs networks; mitigate fragmentation via interoperability, shared procurement, and equitable access.

**Step 4 (years 4-5)**

Conduct multicountry scale-out with RE-AIM (reach, effectiveness, adoption, implementation, and maintenance) evaluation; test sustainable models (eg, subscription-based approaches). This approach mirrors European Union initiatives; mitigate delays via shared contracts and General Data Protection Regulation (GDPR) templates for scalable, equitable VR and AR education.

### Conclusion

Analysis of this international multicountry survey highlights that foundational engagement and confidence are pivotal for promoting advanced adoption of VR and AR technologies and optimizing the use of digital tools. Programs should prioritize an interactive, well-supported, and blended approach to accommodate varying levels of digital familiarity and preferences.

The application of TAM and UTAUT in this study supports their utility in understanding technology adoption in medical education. By addressing perceived usefulness, ease of use, social influence, and facilitating conditions, institutions can design strategies to enhance digital tool integration. In this context, tailored implementation strategies should be developed to optimize digital education adoption. These theoretical advancements inform future research and guide pragmatic policy design aimed at integrating digital tools into postgraduate orthopedic training.

Our findings indicate that digital competence strongly influences acceptance of technology-enhanced education among orthopedic trainees. Therefore, implementation strategies should prioritize competence-building initiatives, such as structured digital literacy programs, as foundational steps. Following competency development, scalable interventions such as VR module integration can be implemented more effectively.

This study advances the understanding of factors influencing digital learning adoption in education, providing a foundation for designing effective, equitable, and innovative learning interventions aligned with trainees’ needs and preferences.

Orthopedic training must advance beyond pandemic-driven digital adoption. While e-learning remains a foundational element, the untapped potential of VR and AR technologies necessitates targeted investments in infrastructure, competency-based curricula, and cross-institutional collaborations. Consistent with the TAM and UTAUT, addressing socioeconomic inequities through policies (such as establishing European Union–funded VR hubs in low-GDP regions) can translate trainee enthusiasm into measurable clinical proficiency.

Beyond educational equity, improving access to high-quality digital and simulation-based training is expected to have downstream clinical relevance, as randomized-trial evidence suggests that simulation training can improve patient-related outcomes in surgery. While our findings are European, the proposed competence-first and equity-oriented implementation pathway could be adapted through partnerships with institutions in the Global South, aligned with World Health Organization priorities for equitable digital health capacity building and the broader global agenda for strengthening surgical systems and workforce development [66,67].

## Appendix

Multimedia Appendix 1 Survey questions.

Multimedia Appendix 2 Elbow and Silhouette methods.

Multimedia Appendix 3 Results of the factor analyses.

Multimedia Appendix 4 R packages.

Multimedia Appendix 5 Geographical distribution of respondents.

Multimedia Appendix 6 Analyses of experience and competence levels.

Multimedia Appendix 7 Results of the questionnaire scores as a function of workplace and gross domestic product (GDP) of the country hosting the training program.

Multimedia Appendix 8 Associations of attitude variables with experience and digital competence as a function of gross domestic product (GDP) category.

Multimedia Appendix 9 Characteristics of low-, medium-, and high–gross domestic product (GDP) countries based on Hofstede’s dimensions of culture.

Multimedia Appendix 10 Detailed description of the operationalization of the suggested research directions.

## Abbreviations

AR: augmented reality
CFI: comparative fit index
EFA: exploratory factor analysis
GDP: gross domestic product
GDPR: General Data Protection Regulation
MI: modification indices
OR: odds ratio
rbs: rank-biserial correlations
RE-AIM: research and reach, effectiveness, adoption, implementation, and maintenance
RMSEA: root mean square error of approximation
SEM: structural equation modeling
SRMR: standardized root mean square residual
TAM: technology acceptance model
TLI: Tucker-Lewis Index
UTAUT: unified theory of acceptance and use of technology
VR: virtual reality

## Data Availability

The datasets generated or analyzed during this study are available in the Mendeley repository [43].

## References

[1] Ogundiya O, Rahman TJ, Valnarov-Boulter I, Young TM (2024). Looking back on digital medical education over the last 25 years and looking to the future: narrative review. J Med Internet Res.

[2] Chowdhury PN, Vaish A, Puri B, Vaishya R (2024). Medical education technology: past, present and future. Apollo Med.

[3] Muttappallymyalil J, Mendis S, John LJ, Shanthakumari N, Sreedharan J, Shaikh RB (2016). Evolution of technology in teaching: blackboard and beyond in medical education. Nepal J Epidemiol.

[4] Han H, Resch DS, Kovach RA (2013). Educational technology in medical education. Teach Learn Med.

[5] Satava RM (1993). Virtual reality surgical simulator: the first steps. Surg Endosc.

[6] Cook DA, Levinson AJ, Garside S, Dupras DM, Erwin PJ, Montori VM (2008). Internet-based learning in the health professions: a meta-analysis. JAMA.

[7] Stirling ERB, Lewis TL, Ferran NA (2014). Surgical skills simulation in trauma and orthopaedic training. J Orthop Surg Res.

[8] Bogár PZ, Tóth Luca, Rendeki S, Mátyus L, Németh N, Boros M, Nagy B, Nyitrai M, Maróti P (2020). [The present and the future of medical simulation education in Hungary]. Orv Hetil.

[9] Cannon WD, Garrett WE, Hunter RE, Sweeney HJ, Eckhoff DG, Nicandri GT, Hutchinson MR, Johnson DD, Bisson LJ, Bedi A, Hill JA, Koh JL, Reinig KD (2014). Improving residency training in arthroscopic knee surgery with use of a virtual-reality simulator: a randomized blinded study. J Bone Joint Surg Am.

[10] Sugand K, Akhtar K, Khatri C, Cobb J, Gupte C (2015). Training effect of a virtual reality haptics-enabled dynamic hip screw simulator. Acta Orthop.

[11] Gani A, Pickering O, Ellis C, Sabri O, Pucher P (2022). Impact of haptic feedback on surgical training outcomes: a randomised controlled trial of haptic versus non-haptic immersive virtual reality training. Ann Med Surg (Lond).

[12] Bogar PZ, Virag M, Bene M, Hardi P, Matuz A, Schlegl AT, Toth L, Molnar F, Nagy B, Rendeki S, Berner-Juhos K, Ferencz A, Fischer K, Maroti P (2024). Validation of a novel, low-fidelity virtual reality simulator and an artificial intelligence assessment approach for peg transfer laparoscopic training. Sci Rep.

[13] Pettinelli NJ, Lee AY, Lee MS, Mahatme RJ, Gillinov SM, Jimenez AE (2023). Virtual reality is an effective tool for learning techniques in arthroplasty: a systematic review and meta-analysis. JAAOS Glob Res Rev.

[14] Cate G, Barnes J, Cherney S, Stambough J, Bumpass D, Barnes CL, Dickinson KJ (2023). Current status of virtual reality simulation education for orthopedic residents: the need for a change in focus. Global Surg Educ.

[15] Dedeilia A, Sotiropoulos MG, Hanrahan JG, Janga D, Dedeilias P, Sideris M (2020). Medical and surgical education challenges and innovations in the COVID-19 era: a systematic review. In Vivo.

[16] Schlégl ÁT, Pintér Z, Kovács A, Kopjár E, Varga P, Kardos D, Gasz B, Füzesi Z (2020). Teaching basic surgical skills using homemade tools in response to COVID-19. Acad Med.

[17] Zhu Q, Liang B, Wang X, Sun X, Wang L (2016). Force–torque intraoperative measurements for femoral shaft fracture reduction. Comput Assist Surg.

[18] Benjamin M, Sabri O (2021). Using haptic feedback in a virtual reality bone drilling simulation to reduce plunge distance. Cureus.

[19] Trute RJ, Alijani A, Erden MS (2024). Visual cues of soft-tissue behaviour in minimal-invasive and robotic surgery. J Robot Surg.

[20] Schostek S, Schurr MO, Buess GF (2009). Review on aspects of artificial tactile feedback in laparoscopic surgery. Med Eng Phys.

[21] von Bechtolsheim F, Bielert F, Schmidt S, Buck N, Bodenstedt S, Speidel S, Lüneburg L-M, Müller T, Fan Y, Bobbe T, Oppici L, Krzywinski J, Dobroschke J, Weitz J, Distler M, Oehme F (2024). Can you feel the force just right? Tactile force feedback for training of minimally invasive surgery-evaluation of vibration feedback for adequate force application. Surg Endosc.

[22] Rangarajan K, Davis H, Pucher PH (2020). Systematic review of virtual haptics in surgical simulation: a valid educational tool?. J Surg Educ.

[23] Shilaskar S, Shinde M, Tadavi S, Shevale P (2024). VR based medical procedure simulator with haptic feedback gloves.

[24] Woodward CJ, Khan O, Aydın A, Dasgupta P, Sinha J (2025). Simulation-based training in orthopedic surgery: a systematic review. Curr Probl Surg.

[25] Harris M, Burke J, Yassin N (2024). Technology-enhanced assessment of surgical training. Bull R Coll Surg Engl.

[26] Salaja B, Feeley A, Feeley I, Sheehan E, Merghani K (2021). Virtual reality simulation in orthopaedic surgical training during periods of restricted clinical hours: a systematic review. J Surg Simul.

[27] McKinney B, Dbeis A, Lamb A, Frousiakis P, Sweet S (2022). Virtual reality training in unicompartmental knee arthroplasty: a randomized, blinded trial. J Surg Educ.

[28] Wolf MA, Pizanis A, Fischer G, Langer F, Scherber P, Stutz J, Orth M, Pohlemann T, Fritz T (2022). COVID-19: a catalyst for the digitization of surgical teaching at a German University Hospital. BMC Med Educ.

[29] Zhao Y, Watterston J (2021). The changes we need: Education post COVID-19. J Educ Chang.

[30] Davis FD (1989). Perceived usefulness, perceived ease of use, and user acceptance of information technology. MIS Quarterly.

[31] Venkatesh, Morris, Davis, Davis (2003). User acceptance of information technology: toward a unified view. MIS Quarterly.

[32] Lewis KO, Cidon MJ, Seto TL, Chen H, Mahan JD (2014). Leveraging e-learning in medical education. Curr Probl Pediatr Adolesc Health Care.

[33] Moore JL, Dickson-Deane C, Galyen K (2011). e-Learning, online learning, and distance learning environments: are they the same?. Internet High Educ.

[34] Tene T, Vique López DF, Valverde Aguirre PE, Orna Puente LM, Vacacela Gomez C (2024). Virtual reality and augmented reality in medical education: an umbrella review. Front Digit Health.

[35] Lee JWY, Tan JY, Bello F (2025). Technology acceptance model in medical education: systematic review. JMIR Med Educ.

[36] Lee AT, Ramasamy RK, Subbarao A (2025). Understanding psychosocial barriers to healthcare technology adoption: a review of TAM technology acceptance model and unified theory of acceptance and use of technology and UTAUT frameworks. Healthcare (Basel).

[37] Soriano GP (2020). Psychometric properties of ‘attitude towards e-learning scale’ among nursing students. Int J Educ Sci.

[38] Özkaya G, Aydin MO, Alper Z (2021). Distance education perception scale for medical students: a validity and reliability study. BMC Med Educ.

[39] Shen S, Xu K, Sotiriadis M, Wang Y (2022). Exploring the factors influencing the adoption and usage of augmented reality and virtual reality applications in tourism education within the context of COVID-19 pandemic. J Hosp Leis Sport Tour Educ.

[40] Artino AR, Phillips AW, Utrankar A, Ta AQ, Durning SJ (2018). "The Questions Shape the Answers": assessing the quality of published survey instruments in health professions education research. Acad Med.

[41] Faul F, Erdfelder E, Lang A, Buchner A (2007). G*Power 3: a flexible statistical power analysis program for the social, behavioral, and biomedical sciences. Behav Res Methods.

[42] Hennig C (2007). Cluster-wise assessment of cluster stability. Comput Stat Data Anal.

[43] Orthopedic and trauma trainees’ attitude towards e-learning and distance learning: an European-wide survey. Mendeley Data.

[44] Soffar A, Elkohail A, Elbanna M, Kassem HA, Murhekar S, Millat MS, Sayed A, Shehata R, Ali Hamoud M, Khalil N (2025). Virtual reality simulation for orthopedic surgical training: a narrative review of current evidence and educational impact. Cureus.

[45] Clarke E (2021). Virtual reality simulation-the future of orthopaedic training? A systematic review and narrative analysis. Adv Simul (Lond).

[46] Gamieldien H, Kruger N, Dey R (2023). MB ChB fifth-year student response to e-learning in orthopaedic surgery during COVID-19. Afr J Health Prof Educ.

[47] Bond M, Buntins K, Bedenlier S, Zawacki-Richter O, Kerres M (2020). Mapping research in student engagement and educational technology in higher education: a systematic evidence map. Int J Educ Technol High Educ.

[48] Elnoor OS, Hundal JS, Chahal US, Kansal SK (2017). Assessment of postgraduate veterinary students’ information literacy competencies and attitude towards e-learning. Indian J Econ Dev.

[49] Langegård U, Kiani K, Nielsen SJ, Svensson P (2021). Nursing students' experiences of a pedagogical transition from campus learning to distance learning using digital tools. BMC Nurs.

[50] Lera M, Taxtsoglou K, Iliadis C, Frantzana A, Kourkouta L (2020). Nurses' attitudes toward lifelong learning via new technologies. Asian Pac Isl Nurs J.

[51] Sommer GM, Broschewitz J, Huppert S, Sommer CG, Jahn N, Jansen-Winkeln B, Gockel I, Hau H-M (2021). The role of virtual reality simulation in surgical training in the light of COVID-19 pandemic: Visual spatial ability as a predictor for improved surgical performance: a randomized trial. Medicine (Baltimore).

[52] Ivanov V, Klygach A, Shterenberg S, Strelkov S, Levy J (2020). Advances in agumented reality (AR) for medical simulation and training. 3C Tecnol Glosas Innov Apl Pyme.

[53] Cianciolo AT, Williams RG, Klamen DL, Roberts NK (2013). Biomedical knowledge, clinical cognition and diagnostic justification: a structural equation model. Med Educ.

[54] Teleki S, Zsidó AN, Lénárd L, Komócsi A, Kiss EC, Tiringer I (2022). Role of received social support in the physical activity of coronary heart patients: the health action process approach. Appl Psychol Health Well Being.

[55] Tick A (2019). An extended TAM Model, for evaluating eLearning acceptance, digital learning and smart tool usage. Acta Polytech Hung.

[56] Cabero-Almenara J, Gutiérrez-Castillo JJ, Guillén-Gámez FD, Gaete-Bravo AF (2022). Digital competence of higher education students as a predictor of academic success. Tech Know Learn.

[57] Bala L, Kinross J, Martin G, Koizia LJ, Kooner AS, Shimshon GJ, Hurkxkens TJ, Pratt PJ, Sam AH (2021). A remote access mixed reality teaching ward round. Clin Teach.

[58] Fidalgo P, Thormann J, Kulyk O, Lencastre JA (2020). Students’ perceptions on distance education: a multinational study. Int J Educ Technol High Educ.

[59] Jan J, Alshare KA, Lane PL (2022). Hofstede’s cultural dimensions in technology acceptance models: a meta-analysis. Univ Access Inf Soc.

[60] Jippes M, Majoor GD (2011). Influence of national culture on the adoption of integrated medical curricula. Adv Health Sci Educ Theory Pract.

[61] Damschroder LJ, Reardon CM, Widerquist MAO, Lowery J (2022). The updated consolidated framework for implementation research based on user feedback. Implement Sci.

[62] Glasgow RE, Harden SM, Gaglio B, Rabin B, Smith ML, Porter GC, Ory MG, Estabrooks PA (2019). RE-AIM planning and evaluation framework: adapting to new science and practice with a 20-year review. Front Public Health.

[63] Cabero-Almenara J, Llorente-Cejudo C, Palacios-Rodríguez A, Gallego-Pérez Ó (2023). Degree of acceptance of virtual reality by health sciences students. Int J Environ Res Public Health.

[64] Safi S, Thiessen T, Schmailzl KJ (2018). Acceptance and resistance of new digital technologies in medicine: qualitative study. JMIR Res Protoc.

[65] Goh J, Clapham M (2014). Attitude to e-learning among newly qualified doctors. Clin Teach.

[66] Meling TR, Meling TR (2021). The impact of surgical simulation on patient outcomes: a systematic review and meta-analysis. Neurosurg Rev.

[67] (2021). Global Strategy on Digital Health 2020-2025.

