# Supplementary material for: e-Learning, Distance Education, and Virtual and Augmented Reality in Orthopedic Training: European Cross-Sectional Survey of Trainee Acceptance Guided by the Technology Acceptance Model and Unified Theory of Acceptance and Use of Technology
Source: JMIR Med Educ. 2026 Jul 10;12:e79418. doi: 10.2196/79418 (PMC13401077; doi:10.2196/79418)
Supplement: Multimedia Appendix 1 [file mededu_v12i1e79418_app1.docx]

## Supplementary material 1. - Survey questions

**Section I – General questions**

1. Age
2. Gender
3. Please rate your English language skills: Beginner/Intermediate/Professional/Mother tongue
4. Country of training program
5. Actual year in speciality training
6. Type/level of your original workplace:
   1. University Clinical Center / National Referral Hospital
   2. Regional / Provincial / County Referral Hospital
   3. City Hospital
   4. Town / Rural Hospital
7. Do you have any previous experience with Distance education
   1. No experience
   2. Heard about it, but never tried
   3. Already tried a few times
   4. Occasional use
   5. Regular use
8. Do you have any previous experience with E-Learning
9. Do you have any previous experience with Medical Simulation
10. Do you have any previous experience with VR/AR Simulation

**Section II – Digital competencies**

1. Do you have any of these digital devices?
   1. PC
   2. Laptop
   3. Smart phone
   4. Tablet
   5. Gaming Console
   6. VR headset
2. Please rate your expertise in use of basic digital tools (From 1 - Non-competent to 5 - Expert)
   Installation of a program or device, data transmission, creation of text files, tabulations (calculations) or presentations etc.
3. Please rate your expertise in use of advanced digital tools
   Programming, web page development, design applications, picture/movie editing etc.
4. Please rate your expertise in use of online tools
   Internet research, online shopping, social networks, online communication etc.
5. Please rate your expertise in digital problem solving
   Helping others with digital problems, set up a network, install operating system etc.
6. Please rate your expertise in use of AR/VR solutions
   AR - Augmented reality, VR - Virtual Reality

**Section III - Attitude towards e-learning scale survey (after Guillasper et al.)**

Generally, "e-learning" refers to the performance of teaching and learning activities using electric media and transfer information, learning materials, and skills through electronic technologies (Akcil et al. 2021).

Likert scale of 5:

1. Strongly disagree
2. Disagree
3. Neutral
4. Agree
5. Strongly agree
6. I am interested in studying courses that utilize e-learning
7. I think that e-learning promotes my learning experiences
8. Presenting courses on the internet makes learning more efficient
9. I intend to use e-learning tools during my training programme if available
10. I am positive about e-learning
11. E-learning environment needs advanced technical knowledge on computer use
12. I would prefer to have courses on the internet rather than in the classroom or face-to-face
13. Online learning is more comfortable and enjoying to me
14. E-learning is a favourable alternative to the pen-paper based system
15. E-learning is not an efficient learning method
16. Over-all, I prefer e-learning and I believe that it is better than traditional method of learning

**We would like to ask shortly your opinion about the use of AR/VR solutions in education (after Shen et al.)**

1. I like the idea of using AR/VR applications in my studies/learning
2. AR/VR applications make learning more interesting
3. I think that would be beneficial to include AR/VR applications in surgical education
4. My general opinion regarding AR/VR applications is positive

**Section IV - Distance education perception scale survey (after Özkaya et al.)**

The term “distance education” refers to the formalized instructional learning where the time/geographic situation constrains learning by not affording in-person contact between student and instructor, or in other words, more generally, it means an educational method which can be characterized with some form of instruction occurs between two parties (a learner and an instructor), it is held at different times and/or places, and uses varying forms of instructional materials (Moore et al. 2011, Lewis et al. 2014).

Likert scale of 5:

1. Strongly disagree
2. Disagree
3. Neutral
4. Agree
5. Strongly agree
6. Diplomas obtained through distance education are as valid as those obtained through face-to-face education
7. Distance education is academically more interesting than face-to-face education
8. The quality of education increases with distance education
9. Programs should be opened in different fields in distance education
10. Distance education is essential to meet the need for trained manpower
11. I believe that in the future, distance education will be more preferred than traditional education
12. Compared to face-to-face education, the cultural diversity of students in distance education is greater
13. My experiences in distance education have positively changed my perspective on distance education
14. In the distance education environment, students get the opportunity to think analytically
15. Student self-control is high in distance education
16. Distance education students socialize more in electronic environment
17. Compared to face-to-face education, distance education provides students with flexibility in terms of resource use
18. Communication tools used in distance education are technologically sufficient
19. Communication tools used in distance education are educationally sufficient
20. Distance education programs are well planned in my training program
21. The learning management system used in the presentation and execution of the courses is sufficient
22. The learning management system used in the presentation, execution and process of the courses is easy to use
23. Students spend less time in distance education than in face-to-face education
24. Compared to face-to-face education, distance education provides students with flexibility in terms of time usage
25. Training centers give students access to electronic material to support distance education
26. Training centers prepare electronic materials such as e-books and e-journals to support distance education for students
27. Students are provided with sufficient technical support to solve technical problems they encounter in distance education
