# Supplementary material for: e-Learning, Distance Education, and Virtual and Augmented Reality in Orthopedic Training: European Cross-Sectional Survey of Trainee Acceptance Guided by the Technology Acceptance Model and Unified Theory of Acceptance and Use of Technology
Source: JMIR Med Educ. 2026 Jul 10;12:e79418. doi: 10.2196/79418 (PMC13401077; doi:10.2196/79418)
Supplement: Multimedia Appendix 2 [file mededu_v12i1e79418_app2.docx]

## Supplementary material 2 – Elbow and Silhouette methods


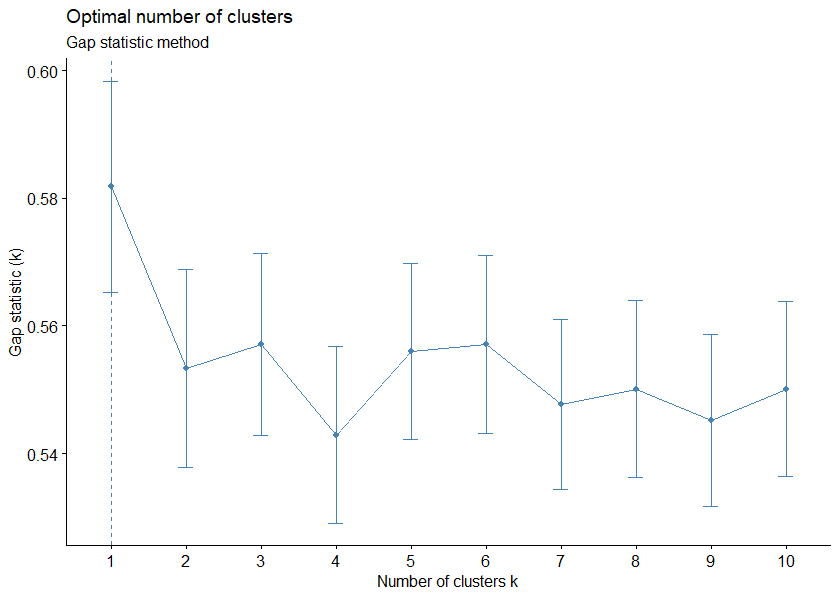


**Supplementary Figure 2.1.** Testing the optimal number of k-means clusters using gap statistic


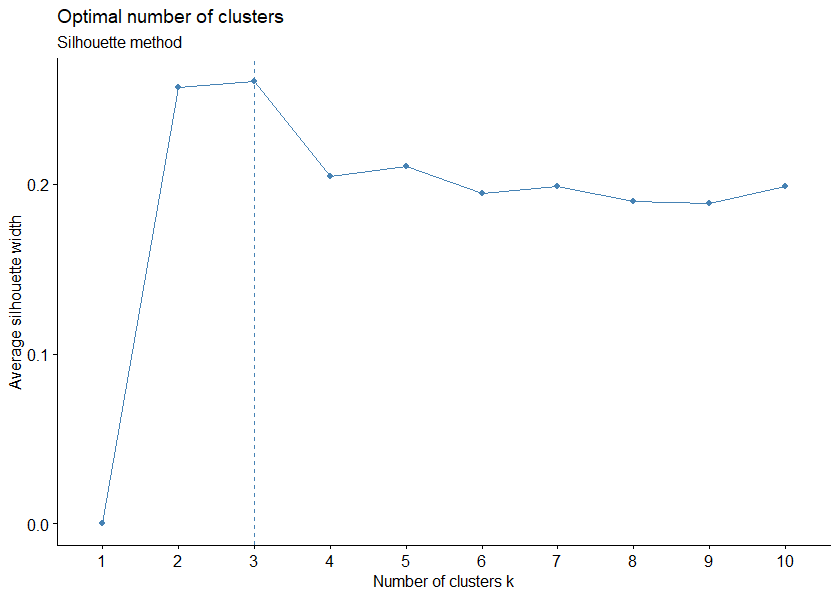


**Supplementary Figure 2.2.** Testing the optimal number of k-means clusters using Silhouette method


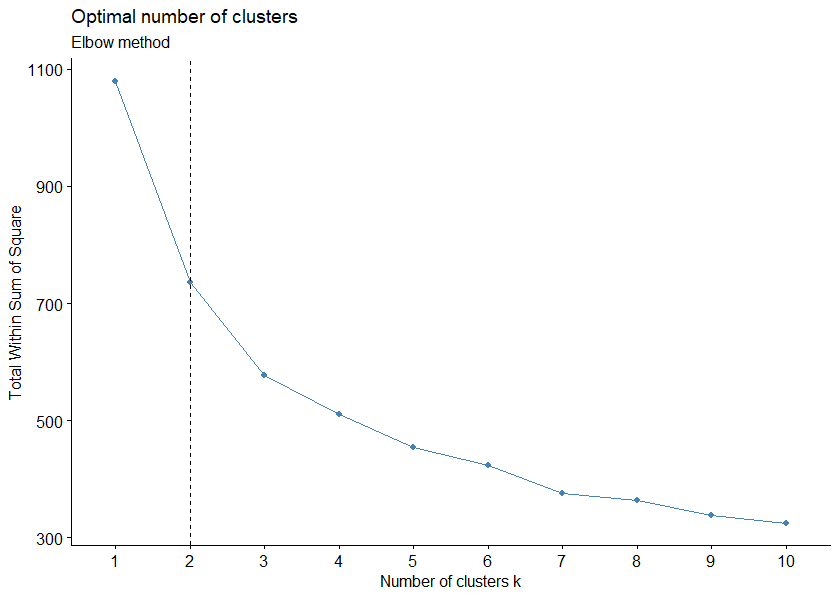


**Supplementary Figure 2.3.** Testing the optimal number of k-means clusters using the elbow method

**Choosing the final number of clusters**

The Gap statistic suggested that the optimal number of k-means clusters was one. The Silhouette method indicated three clusters, whereas the elbow method suggested two clusters. In selecting the final number of clusters, we considered both theoretical relevance and a comparison of the abovementioned methods. A one-cluster solution was deemed theoretically meaningless. To decide between the two- and three-cluster solutions, the Gap statistic was examined in more detail. This analysis indicated that the three-cluster solution was slightly superior and was therefore retained for subsequent analyses


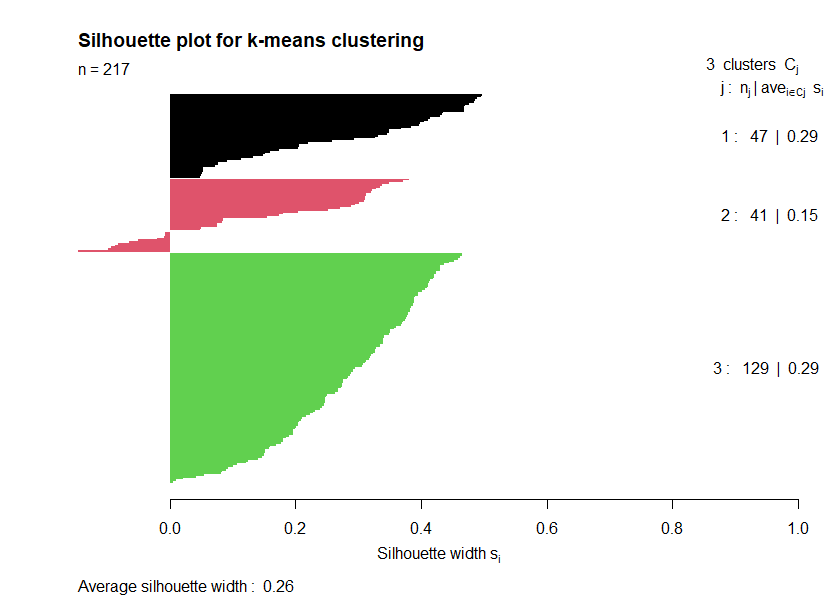


**Supplementary Figure 2.4.** Silhouette plot for the three-cluster solution
