# Supplementary material for: e-Learning, Distance Education, and Virtual and Augmented Reality in Orthopedic Training: European Cross-Sectional Survey of Trainee Acceptance Guided by the Technology Acceptance Model and Unified Theory of Acceptance and Use of Technology
Source: JMIR Med Educ. 2026 Jul 10;12:e79418. doi: 10.2196/79418 (PMC13401077; doi:10.2196/79418)
Supplement: Multimedia Appendix 3 [file mededu_v12i1e79418_app3.docx]

## Supplementary material 3 - Results of the Factor Analyses

**Supplementary Materials for Exploratory Factor Analysis**

The exploratory factor analysis, conducted on the survey data from orthopaedic trainees, utilized a polychoric correlation matrix to appropriately handle the ordinal data derived from Likert-scale responses. As stated in the main text, inter-item correlations were generally moderate (median rₛ = .368, range = 0 – 0.925). One pair of items showed a very high correlation (rₛ = .925). These items were retained because they assess theoretically distinct but related constructs -attitudes toward VR/AR use in education broadly versus in surgical education specifically. The high association is therefore interpreted as reflecting conceptual proximity rather than redundancy. Aside from this pair, only seven correlations exceeded .80, suggesting limited risk of item redundancy or local dependence within the scale.

Parallel analysis recommended the retention of six distinct factors which, together, describe a substantial proportion of the variability observed in the responses concerning digital learning modalities.


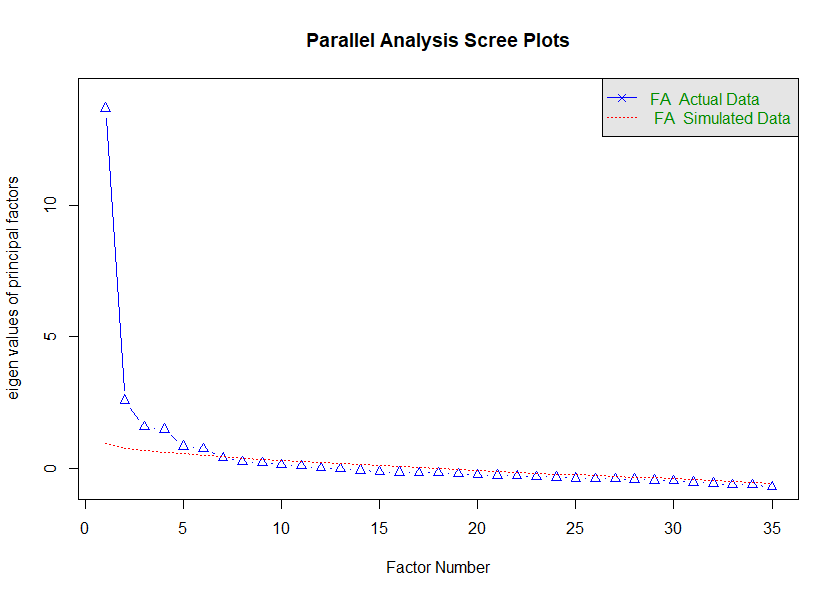


**Supplementary Figure 3.1.** Scree Plot from Parallel Analysis of Factor Analysis.

Scree plot demonstrating the eigenvalues from actual survey data compared to those generated from simulated data in a parallel analysis. The plot is used to determine the number of factors to retain for the exploratory factor analysis of orthopaedic trainees' survey data. Each point represents an eigenvalue associated with a factor number, with the solid line (triangles) representing the eigenvalues from the actual survey data and the dashed line showing the eigenvalues from simulated random data. The plot suggests a clear drop after the seventh factor, indicating that seven factors sufficiently capture the underlying structure of the data without overfitting.

As explained in the main text, theory-driven model refinement resulted in a parsimonious four-factor solution with the factors Distance Education Perception (DEP), Equipment Facility Perception (EFP), Attitude Toward AR/VR (ATA), and Attitude Toward E-learning (ATE).

**Factorial composition of the initial model including all variables:**

1. **Distance Education Perception (DEP):** Distance Education items #1 - #11 as well as four E-learning items (#7-9, 11) loaded on this factor, with loadings ranging from 0.351 to 0.927.
2. **Equipment Facility Perception (EFP):** This factor was loaded exclusively by Distance Education items #12-17 and #20-22. Factor loadings ranged between 0.413 and 0.831.
3. **Attitude Toward AR/VR (ATA):** all VR/AR-related items loaded on the same factor with loadings ranging from 0.835 to 0.937.
4. **Attitude Toward E-learning (ATE):** this factor included E-learning items #1-5 and #10. Loadings ranged between -0.509 and 0.806.

Only one item (E-learning #6) did not load on any factor.

**Supplementary Figure 3.2. Item communalities**

*Note: Red horizontal line indicates the item communality criterion.*

**Visualization of Factor Loadings:** The heatmap visualization underscored the robustness of the factor loadings, clearly delineating the contribution of each survey item to the respective factors. The color gradient effectively highlighted the strength of associations, aiding in the identification of cross-loadings and items with weaker contributions.

**Implications and Applications:** These findings underscore the importance of various facets of digital learning in orthopedic training. Specifically, the distinct factors identified can inform targeted enhancements in educational strategies and training curricula, ensuring that they align closely with trainee preferences and educational needs, particularly in the rapidly evolving domains of e-learning and VRAR.


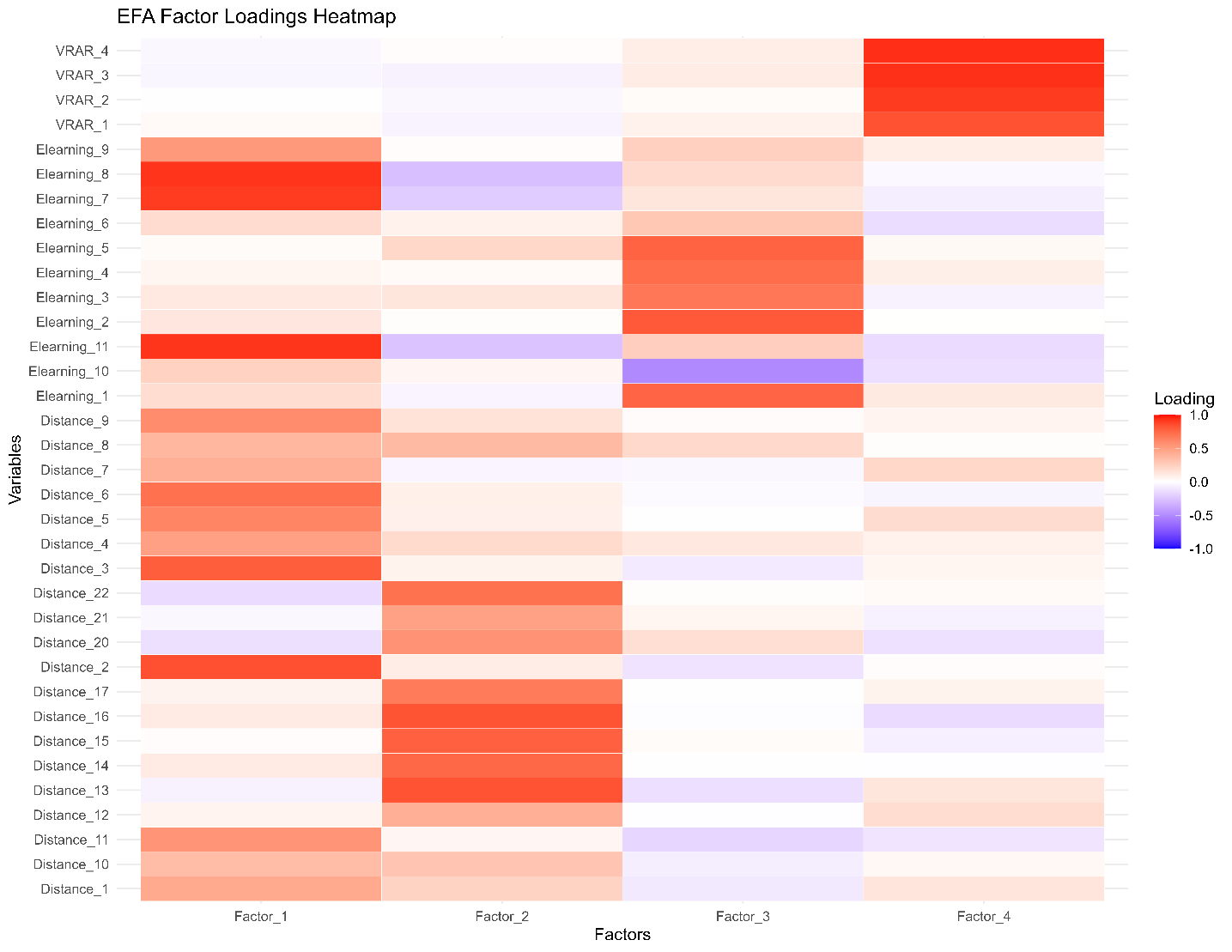


**Supplementary Figure 3.3.** Factor Loadings Heatmap for Orthopaedic Trainees' Attitudes Towards Digital Learning Modalities.

Heatmap displaying the factor loadings from an exploratory factor analysis of survey responses from orthopaedic trainees. Each row represents a survey item, and each column corresponds to one of four extracted factors. The colour intensity reflects the strength and direction of the loading, with red indicating strong positive associations, blue indicating strong negative associations, and white representing no association. This visualization highlights the distinct patterns of association between survey items and factors, elucidating the underlying dimensions of trainees' attitudes towards various aspects of digital learning, including virtual reality/augmented reality (VRAR), e-learning, and distance education facilities.

**Supplementary Materials for Confirmatory Factor Analysis**

Guided by the EFA results, a confirmatory factor analysis (CFA) was performed on 22 items specified to load on four factors. The initial model was refined in multiple steps based on the suggestions of modification indices. The changes in the factor structure as well as the resulted model improvements are presented in Supplementary Table 3.1.

**Supplementary Table 3.1. Changes in model fit indices in three steps of model refinement.**

| **Model** | **Change** | **X^2^** | **df** | **CFI** | **TLI** | **AIC** | **RMSEA** | **SRMR** |
| --- | --- | --- | --- | --- | --- | --- | --- | --- |
| Initial model | - | 370.573 | 203 | 0.938 | 0.93 | 10840.7 | 0.062 | 0.052 |
| Refined 1 | Distance_13  ~~ Distance_14 | 342.65 | 202 | 0.948 | 0.941 | 10810.8 | 0.057 | 0.054 |
| Refined 2 (final) | Elearning_1  ~~ Elearning_2 | 323.203 | 201 | 0.955 | 0.948 | 10791 | 0.053 | 0.053 |
| Refined 3 | Distance_2  ~~ Distance_3 | 311.08 | 200 | 0.959 | 0.953 | 10778.9 | 0.051 | 0.052 |

In the final CFA model, two residual covariances were allowed to correlate: DE items #13 and #14, E-learning items #1 and #2. The correlation between DE items #13 (“*Communication tools used in distance education are technologically sufficient*”) and #14 (“*Communication tools used in distance education are educationally sufficient*”) was allowed because these items are very similarly worded since there is only one word that is different. The residual covariance between E-learning items #1 (“*I am interested in studying courses that utilize e-learning*”) and #2 (“*I think that e-learning promotes my learning experiences*”) was also freely estimated because both items reflect closely related evaluative judgments about e-learning (interest and perceived benefit). Their semantic similarity and shared evaluative framing likely induce local dependence beyond the latent attitude factor.
