# Supplementary material for: e-Learning, Distance Education, and Virtual and Augmented Reality in Orthopedic Training: European Cross-Sectional Survey of Trainee Acceptance Guided by the Technology Acceptance Model and Unified Theory of Acceptance and Use of Technology
Source: JMIR Med Educ. 2026 Jul 10;12:e79418. doi: 10.2196/79418 (PMC13401077; doi:10.2196/79418)
Supplement: Multimedia Appendix 4 [file mededu_v12i1e79418_app4.docx]

## Supplementary material 4 – R packages

Within R, the following packages were used for specific analytical procedures:

- ‘psych’ for polychoric correlations, EFA, and reliability indices [1];
- ‘lavaan’ for CFA[2];
- ‘semTools’ and ‘semPlot’ for model evaluation and graphical visualization of standardized path coefficients [3,4]; and
- ‘fpc’ for cluster stability assessment using Jaccard similarity values and ‘cluster’ for determining the optimal number of clusters [5,6]

1. Revelle W, Revelle MW. Package ‘Psych.’ *The Comprehensive R Archive Network*. Published online 2015.

2. Rosseel Y. lavaan An R Package for Structural Equation Modeling. *J Stat Softw*. 2012;48(2). doi:10.18637/jss.v048.i02

3. Jorgensen TD, Pornprasertmanit S, Schoemann AM, Rosseel Y. semTools: Useful Tools for Structural Equation Modeling. *CRAN: Contributed Packages*. Preprint posted online May 18, 2012. doi:10.32614/CRAN.package.semTools

4. Epskamp S. semPlot: Path Diagrams and Visual Analysis of Various SEM Packages’ Output. *CRAN: Contributed Packages*. Preprint posted online January 28, 2013. doi:10.32614/CRAN.package.semPlot

5. Hennig Christian. https://CRAN.R-project.org/package=fpc. 2024.

6. Maechler M, Rousseeuw P, Struyf A, Hubert M. cluster: “Finding Groups in Data”: Cluster Analysis Extended Rousseeuw et al. *CRAN: Contributed Packages*. Preprint posted online April 12, 1999. doi:10.32614/CRAN.package.cluster
