# Supplementary material for: e-Learning, Distance Education, and Virtual and Augmented Reality in Orthopedic Training: European Cross-Sectional Survey of Trainee Acceptance Guided by the Technology Acceptance Model and Unified Theory of Acceptance and Use of Technology
Source: JMIR Med Educ. 2026 Jul 10;12:e79418. doi: 10.2196/79418 (PMC13401077; doi:10.2196/79418)
Supplement: Multimedia Appendix 5 [file mededu_v12i1e79418_app5.docx]

## Supplementary material 5. - Geographical distribution of respondents


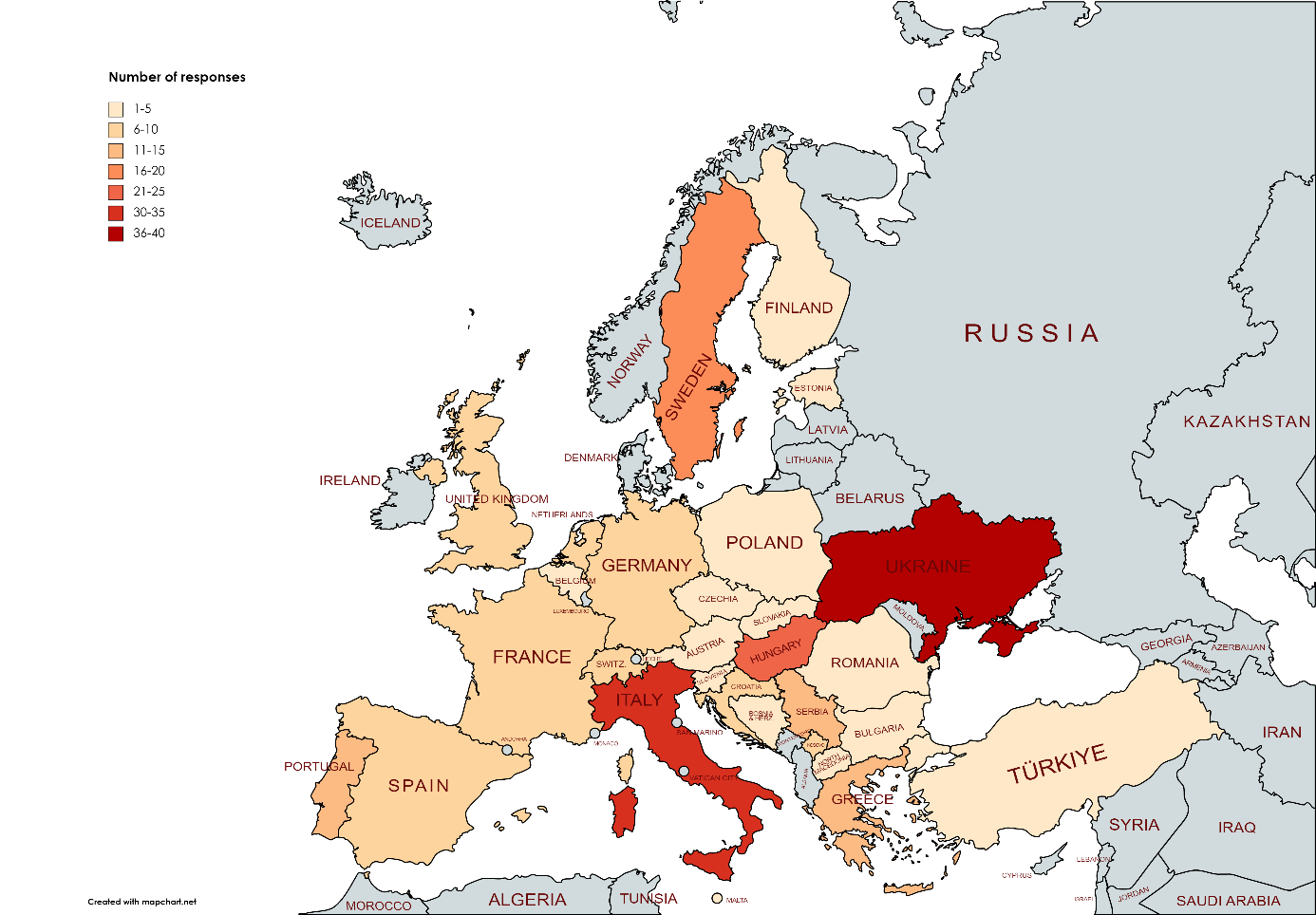


**Supplementary Figure 5.1.** Geographical distribution of respondents (created with mapchart.net)

Map lines delineate study areas and do not necessarily depict accepted national boundaries.
