# Supplementary material for: e-Learning, Distance Education, and Virtual and Augmented Reality in Orthopedic Training: European Cross-Sectional Survey of Trainee Acceptance Guided by the Technology Acceptance Model and Unified Theory of Acceptance and Use of Technology
Source: JMIR Med Educ. 2026 Jul 10;12:e79418. doi: 10.2196/79418 (PMC13401077; doi:10.2196/79418)
Supplement: Multimedia Appendix 6 [file mededu_v12i1e79418_app6.docx]

## Supplementary material 6 - Analyses of Experience and Competence Levels

### 6.1 Experience with e-learning

The participants reported their levels of experience across four domains: distance education, e-learning, medical simulation, and virtual/augmented reality (VR/AR).

For distance education, "Occasional Use" was the most commonly reported level of experience (28.1%), followed closely by "Regular Use" (25.3%) and "Tried a Few Times" (24.0%). A smaller proportion of participants reported "No Experience" (12.4%) or "Heard About, Never Used" (10.1%). The mean experience level was 3.44 (median = 4), with the mode corresponding to "Occasional Use". E-learning exhibited the highest overall adoption among the four domains. "Occasional Use" was the most frequently reported experience level (34.6%), with a substantial proportion also reporting "Regular Use" (25.8%) and "Tried a Few Times" (27.6%). Only a minority of participants reported "No Experience" (6.5%) or "Heard About, Never Used" (5.5%). The mean experience level was 3.68 (median = 4), and the mode was "Occasional Use."

For medical simulation, responses were concentrated at intermediate levels of experience. "Tried a Few Times" was the most frequent response (44.2%), followed by "Heard About, Never Used" (20.7%) and "Occasional Use" (18.0%). Fewer participants reported "No Experience" (12.9%) or "Regular Use" (4.2%). The mean experience level was 2.80 (median = 3), with "Tried a Few Times" being the modal level. Experience with VR/AR was notably lower compared to the other domains. "No Experience" (29.0%) and "Heard About, Never Used" (38.2%) dominated the responses, while "Tried a Few Times" (23.5%) and "Occasional Use" (6.9%) were less common. Only 2.3% of participants reported "Regular Use." The mean experience level was 2.15 (median = 2), with the mode being "Heard About, Never Used."


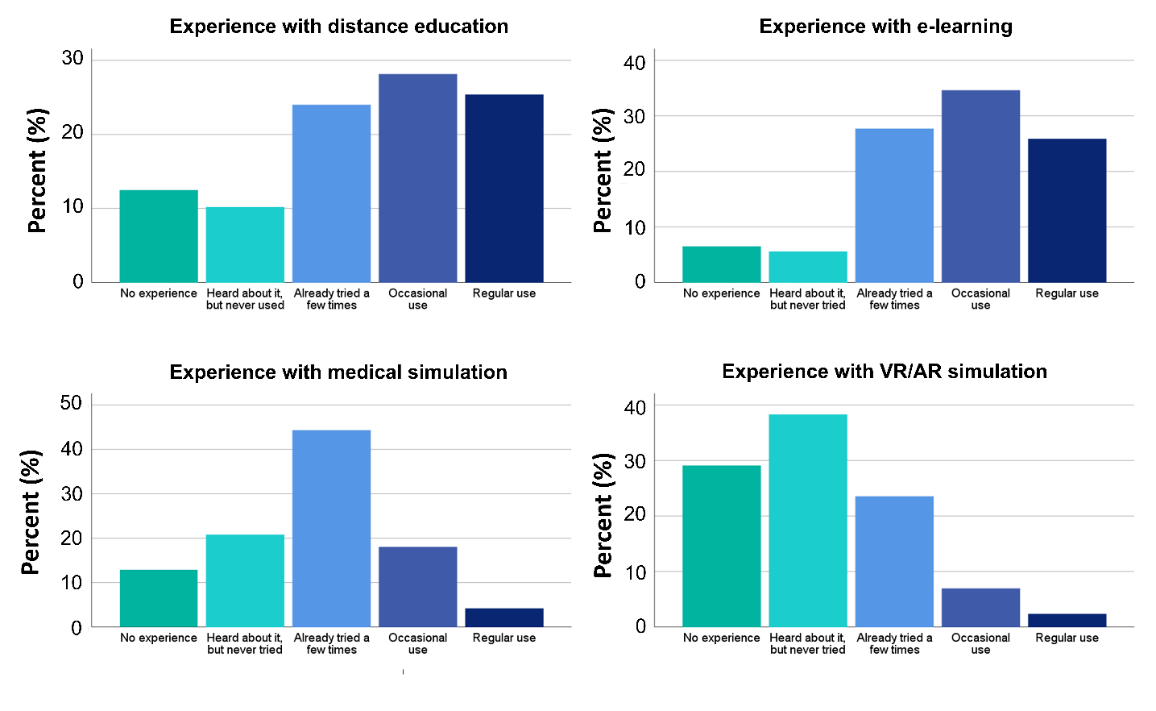


**Supplementary Figure 6.1.** Distribution of responses to the questions regarding experience with distance education, e-learning, medical simulation, and Virtual/Augmented Reality simulation

**
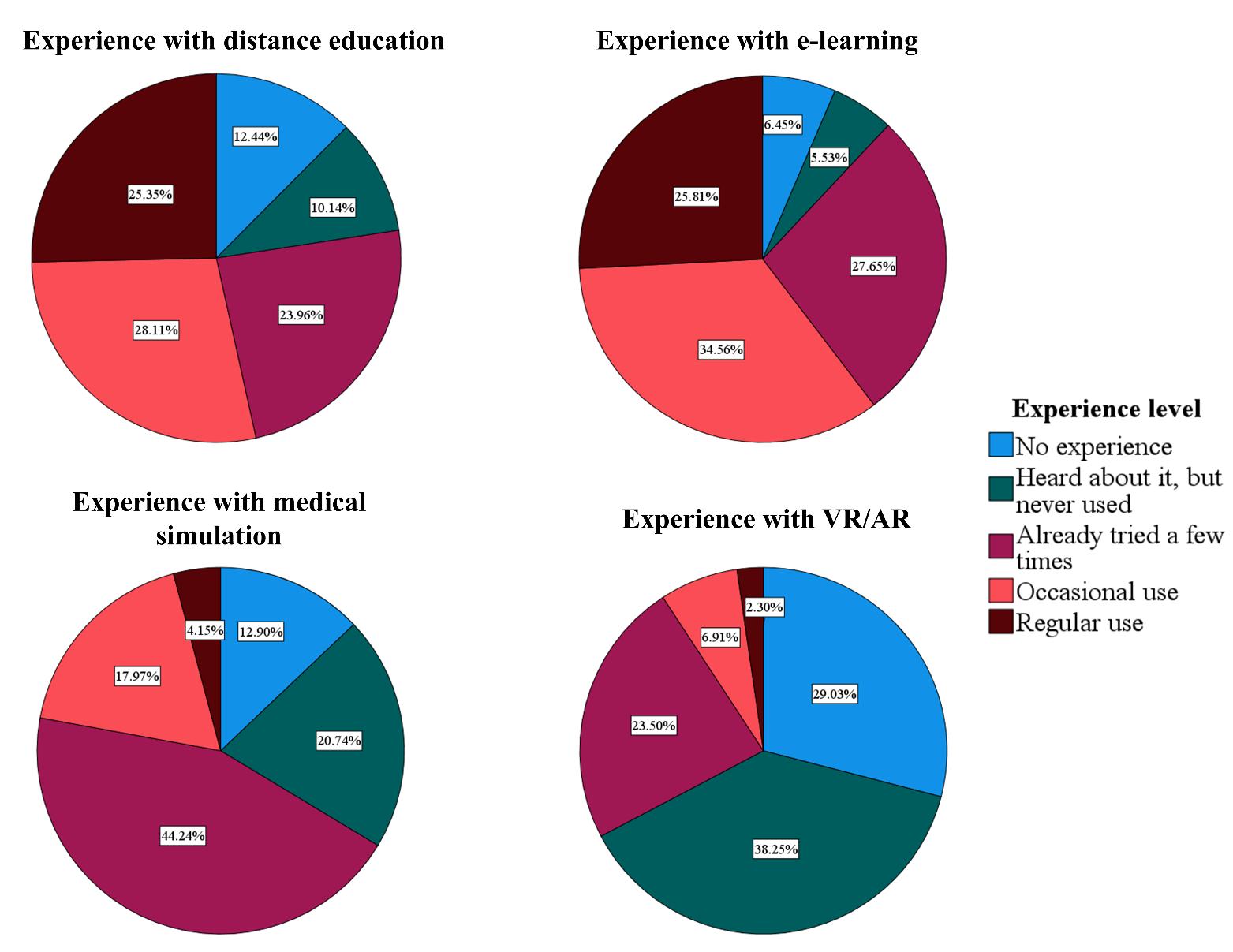
Supplementary Figure 6.2.** Distribution of responses to questions about experience with distance learning and e-learning

### 6.2 Digital competence

The assessment of digital competence among participants revealed substantial variation in proficiency across different domains, reflecting both areas of strength and opportunities for skill enhancement. The findings, summarized in Table 2, highlight a dichotomy between foundational digital skills, where competence was overwhelmingly high, and advanced or emerging technologies, where gaps were more pronounced.

#### 6.2.1 Core Digital Skills

Competence with basic digital tools, such as data transmission and text file creation, was universally high, with nearly 78% of participants identifying as having "Advanced skills" or "Expert" proficiency. Similarly, proficiency with online tools, including internet research and communication platforms, was exceptionally robust; 82.5% of participants rated their abilities as "Advanced" or "Expert." Notably, no participants reported a complete lack of competence in these domains. This consistency underscores the ubiquity and accessibility of these skills in modern education and professional environments.

#### 6.2.2 Advanced Tools and Problem Solving

In contrast, proficiency with advanced digital tools, such as programming and video editing, displayed a wider spread. While 24.9% of participants rated themselves as having "Intermediate skills," a significant proportion (21.2%) reported being "Not competent at all." Similarly, digital problem-solving, which involves tasks like setting up networks or assisting others with technical issues, exhibited a broad distribution of responses. Here, "Intermediate" and "Advanced skills" were the most common (30% and 27.2%, respectively), but approximately one-third of participants rated themselves at the "Basic skills" level or lower. These findings suggest that while some respondents excel in higher-level digital capabilities, others experience limitations, potentially impacting their ability to adapt to evolving technological demands.

#### 6.2.3 VR/AR Solutions

The domain of VR/AR solutions stood out for its significant proportion of respondents (47.5%) reporting no competence. Only 11.1% of participants rated their proficiency at "Advanced" or "Expert" levels, reflecting a substantial gap in familiarity and usage of these emerging technologies. This contrast with the high proficiency reported in other areas highlights VR/AR as an area requiring targeted training and support to build capacity.

#### 6.2.4 Relationships Among Competence Domains

The data suggests a continuum of digital competence, with foundational skills forming a baseline for more specialized capabilities. Participants proficient in basic and online tools were more likely to exhibit advanced skills in digital problem-solving and, to a lesser extent, advanced tools. However, the inverse was not universally true, indicating that foundational competence is necessary but not sufficient for mastery of specialized or emerging technologies.

The low levels of competence in VR/AR solutions, even among those adept at other digital skills, suggest that familiarity with emerging technologies depends on distinct experiences and exposure, rather than building directly from general digital competence. This gap highlights the need for dedicated interventions to bridge the divide between traditional digital skills and emerging technological proficiencies.

| **Digital competence** | *Not competent at all (%)* | *Basic skills (%)* | *Inter-mediate skills (%)* | *Advanced skills (%)* | *Expert (%)* |
| --- | --- | --- | --- | --- | --- |
| Basic digital tools (e.g., installation, data transmission, creation of text files) | 0 (0) | 10 (4.6) | 38 (17.5) | 101 (46.5) | 68 (31.3) |
| Advanced digital tools (e.g., programming, movie editing) | 46 (21.2) | 63 (29) | 54 (24.9) | 38 (17.5) | 16 (7.4) |
| Online tools (e.g., internet research, online shopping/communication) | 0 (0) | 6 (2.8) | 32 (14.7) | 94 (43.3) | 85 (39.2) |
| Digital problem solving (e.g., setting up a network, helping others with digital problems) | 25 (11.5) | 45 (20.7) | 65 (30) | 59 (27.2) | 23 (10.6) |
| VR/AR solutions | 103 (47.5) | 54 (24.9) | 36 (16.6) | 16 (7.4) | 8 (3.7) |

**Supplementary Table 6.1.** Distribution of answers to digital competence-related questions

**
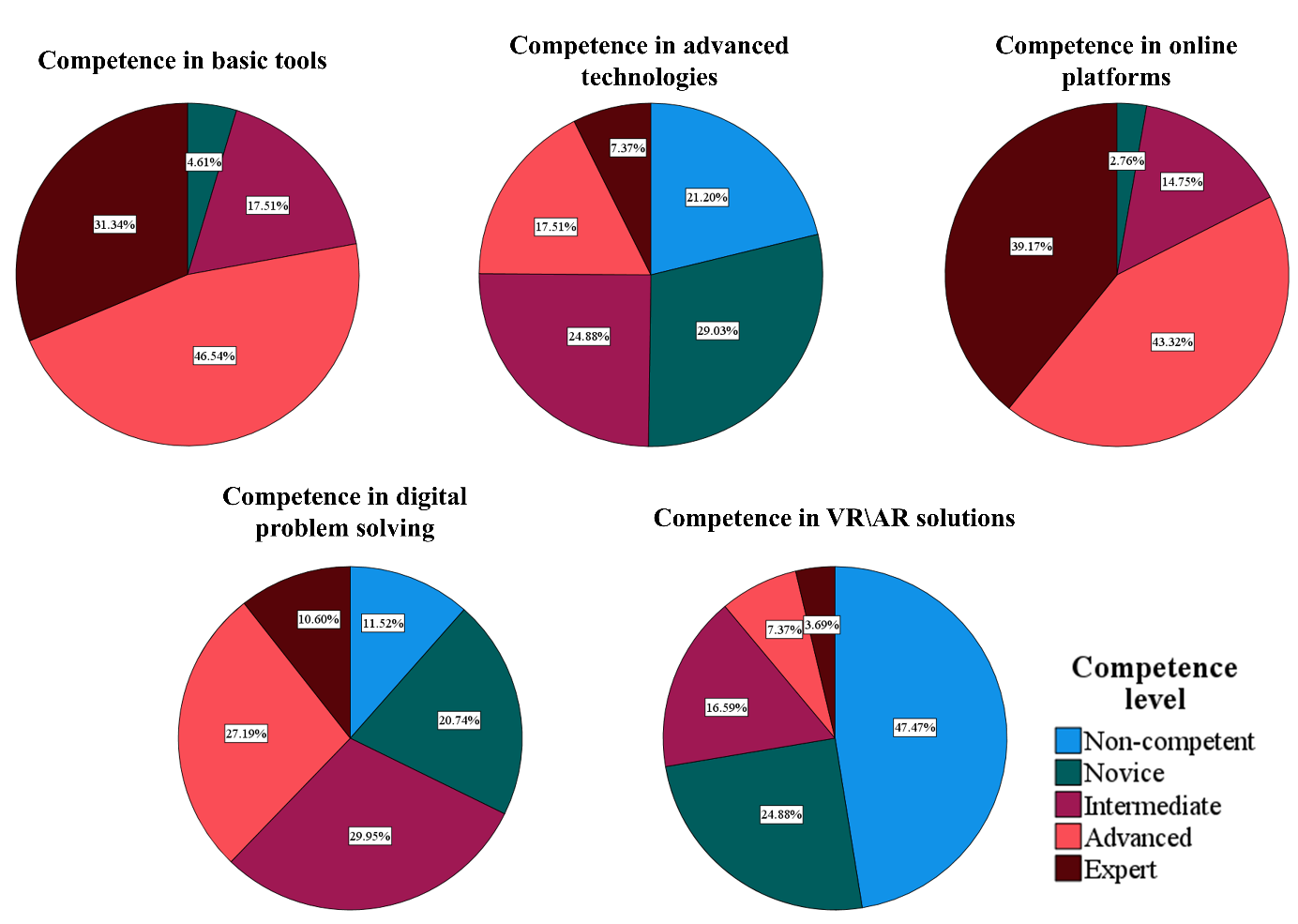
**

**Supplementary Figure 6.3.** Distribution of responses to questions about digital competence
