# Supplementary material for: e-Learning, Distance Education, and Virtual and Augmented Reality in Orthopedic Training: European Cross-Sectional Survey of Trainee Acceptance Guided by the Technology Acceptance Model and Unified Theory of Acceptance and Use of Technology
Source: JMIR Med Educ. 2026 Jul 10;12:e79418. doi: 10.2196/79418 (PMC13401077; doi:10.2196/79418)
Supplement: Multimedia Appendix 7 [file mededu_v12i1e79418_app7.docx]

## Supplementary material 8 - Results of the questionnaire scores as a function of workplace and GDP of the country hosting the training program.


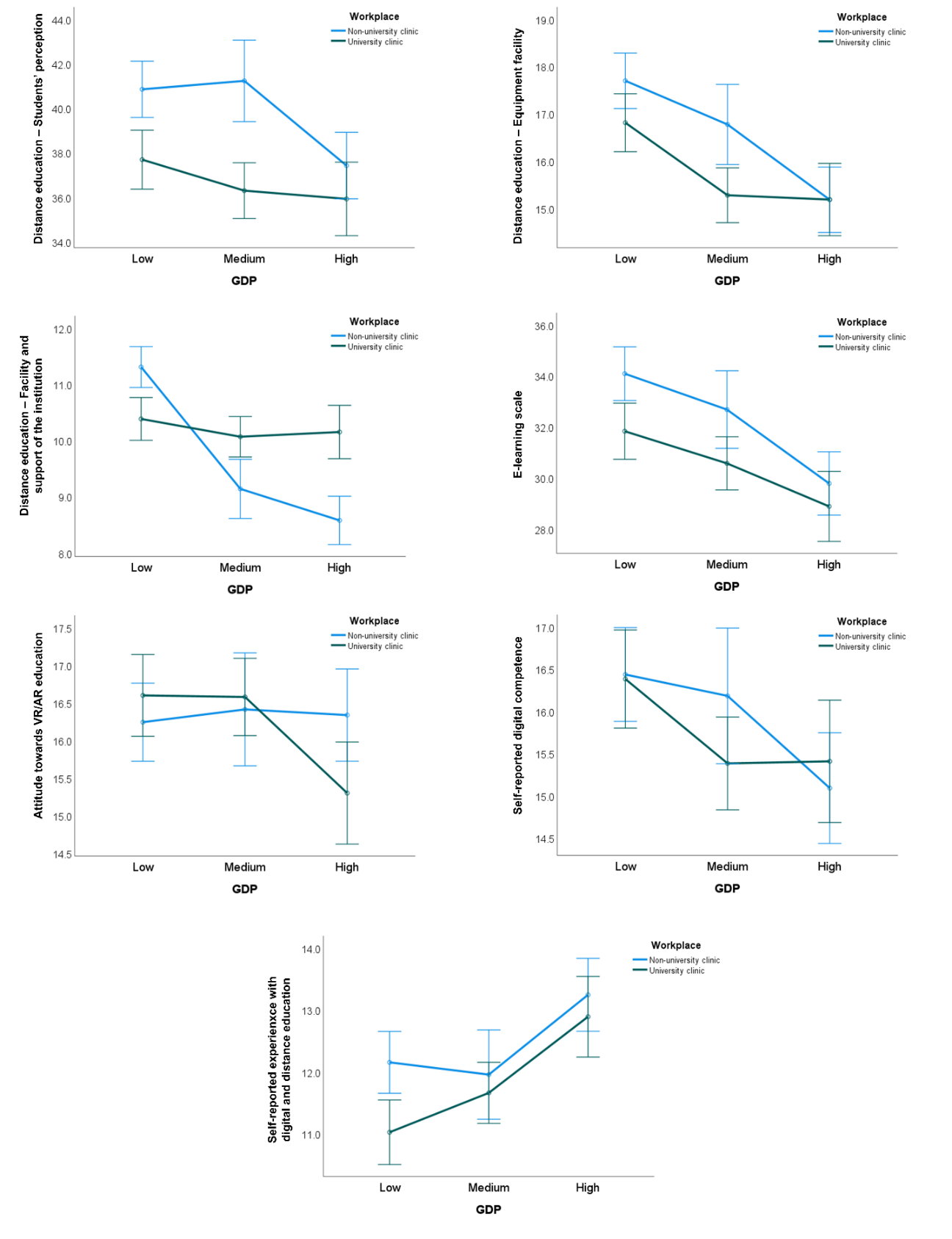


**Supplementary Figure 8.1.** Error bars represent ± 1 standard error. Low GDP countries: GDP per capita is lower than 19,000 USD, medium: 19–40,000 USD, high: above 40,000 USD
