# Supplementary material for: e-Learning, Distance Education, and Virtual and Augmented Reality in Orthopedic Training: European Cross-Sectional Survey of Trainee Acceptance Guided by the Technology Acceptance Model and Unified Theory of Acceptance and Use of Technology
Source: JMIR Med Educ. 2026 Jul 10;12:e79418. doi: 10.2196/79418 (PMC13401077; doi:10.2196/79418)
Supplement: Multimedia Appendix 8 [file mededu_v12i1e79418_app8.docx]

## Supplementary material 7 - Associations of attitude variables with experience and digital competence as a function of GDP category.

**
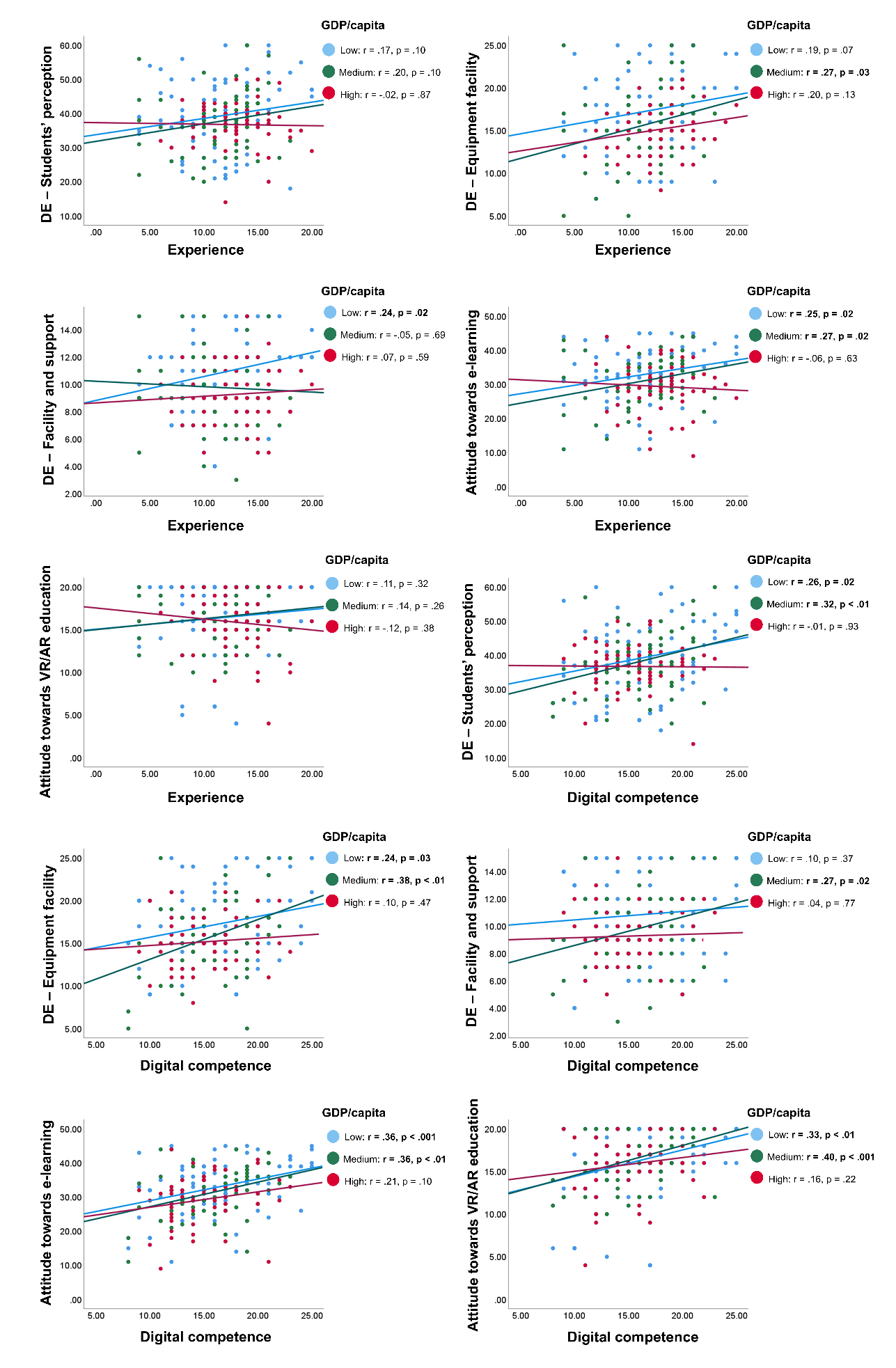
**

**Supplementary Figure 7.1.** Significant associations are written in bold. Abbreviations: DE = distance education
