# Supplementary material for: e-Learning, Distance Education, and Virtual and Augmented Reality in Orthopedic Training: European Cross-Sectional Survey of Trainee Acceptance Guided by the Technology Acceptance Model and Unified Theory of Acceptance and Use of Technology
Source: JMIR Med Educ. 2026 Jul 10;12:e79418. doi: 10.2196/79418 (PMC13401077; doi:10.2196/79418)
Supplement: Multimedia Appendix 9 [file mededu_v12i1e79418_app9.docx]

## Supplementary material 9 – Characteristics of the Low/Medium/High GDP countries based on the Hofstede dimensions of culture

Source: <https://www.theculturefactor.com/> (Accessed: 2026.02.08.)

**Supplementary Table 9.1**. Characteristics of the Low/Medium/High GDP countries based on the Hofstede dimensions of culture
The average values of the GDP per capita groups we defined (low <29,000 USD, medium 29,000–40,000 USD and high >40,000 USD).
*No data found for Republic of Kosovo

| **Dimension** | **GDP per capita** | | |
| --- | --- | --- | --- |
|  | **Low** | **Medium** | **High** |
| n (country) | 8* | 10 | 10 |
| Power distance | 78.38 | 66.88 | 45.00 |
| Individualism | 46.75 | 61.25 | 78.88 |
| Motivation towards Achievement and Success | 43.13 | 52.00 | 40.63 |
| Uncertainty Avoidance | 88.88 | 81.13 | 63.75 |
| Long Term Orientation | 42.00 | 48.63 | 56.50 |
| Indulgence | 33.63 | 31.13 | 63.63 |
