# Supplementary material for: e-Learning, Distance Education, and Virtual and Augmented Reality in Orthopedic Training: European Cross-Sectional Survey of Trainee Acceptance Guided by the Technology Acceptance Model and Unified Theory of Acceptance and Use of Technology
Source: JMIR Med Educ. 2026 Jul 10;12:e79418. doi: 10.2196/79418 (PMC13401077; doi:10.2196/79418)
Supplement: Multimedia Appendix 10 [file mededu_v12i1e79418_app10.docx]

## Supplementary material 10 – Detailed description of the operationalization of the suggested research directions

To operationalize the suggested research directions and translate them into actionable policy and funding pathways, we propose a scalable four-step approach aligned with current European Union funding schemes and developed in collaboration with international professional organizations and networks. To maintain feasibility, each step includes time-bound milestones and explicit implementation risks/mitigations, consistent with determinant-based implementation frameworks (e.g., CFIR) and impact/sustainability evaluation (e.g., RE-AIM and the Dynamic Sustainability Framework).

Step 1. The first phase aims to generate early evidence on feasibility, acceptability, and educational value. *Year 1* pilots across **3–5 training centres** with an indicative cohort of **~30-50 trainees**, with predefined feasibility endpoints (e.g., recruitment/completion rates, usability/acceptability, minimum technical requirements, and faculty time burden). Pilot projects should focus on AR/VR-based international training for orthopedic and trauma trainees using existing EU funding. ERASMUS+ (Key Actions 2 and 3) can support curriculum development, faculty training, and cross-border programs. Erasmus+ Small-scale Partnerships (typically 6–24 months) can support rapid proof-of-concept curriculum development, faculty training, and cross-border delivery Horizon Europe (Cluster 1: Health and Cluster 4: Digital) can fund technology development, system improvement, and translational clinical validation; planning should account for the evaluation period that can take up to ~5 months EU4Health can support workforce upskilling, while EIT Health can test scalable digital solutions. Key risks include heterogeneous baseline infrastructure, limited protected faculty time, and unequal access; mitigations include minimum device specifications, train-the-trainer packages, staged onboarding, and early determinant mapping to target site-specific barriers.

Step 2. *Years 2–3* multicenter comparative studies (preferably preregistered), with an indicative target of ≥150 trainees aggregated across sites, and standardized outcomes (e.g., OSATS, SCORE), alongside time/resource efficiency, cost per trainee, and return on investment. A formal cost–benefit analysis should conclude this phase. Potential risk factors are contamination between study arms, variable supervision intensity, and inconsistent assessment; the mitigations include cluster designs where feasible, harmonized assessor training, and a core outcome set with site-level adjustment.

Step 3. The goal is to establish regional AR/VR competency hubs that support training, content sharing, and faculty development. Years 3–4 is about the establishment of 2–4 regional hubs with (a) a shared modular curriculum, (b) a faculty development pathway, and (c) an access model that explicitly includes low-resource sites (e.g., shared access or lending Scaling should use existing European simulation and innovation ecosystems. SESAM already connects simulation centers and can standardize curriculum, faculty development, and quality assurance. European Digital Innovation Hubs (Digital Europe Programme) provide technical infrastructure, AI integration, and digital skills training, allowing AR/VR hubs to be embedded within the EDIH network. Platform fragmentation and widening inequality may occour and cause challenge; but including interoperability requirements, shared procurement models, and equity targets (e.g., reserved trainee slots and subsidized access for low-resource centers) can mitigate these risks.

Step 4. This phase should conduct coordinated multi-country studies to validate AR/VR training across diverse healthcare systems, assess adaptability to local socioeconomic contexts, and ensure equitable access in both high- and low-resource settings. Years 4–5 scale-out with explicit evaluation of reach, adoption, implementation quality, and maintenance (RE-AIM), and a sustainability plan that anticipates ongoing adaptation and improvement (Dynamic Sustainability Framework) Sustainable models (e.g. shared platforms, subscriptions, cross-institution licenses) should be tested. This mirrors successful EU initiatives such as European Reference Networks, aviation simulation networks, and cross-border cybersecurity centers. In this last phase, the risks include procurement delays, device maintenance burden, and data governance constraints; mitigations include staged onboarding with technical support, shared service/maintenance contracts, and GDPR-aligned governance template. The goal is a scalable, equitable, and sustainable model for AR/VR-based medical education across Europe.
